# Data‐Driven Characterization of Knee Structures Using Non‐Negative Matrix Factorization of 3D Multi‐Echo UTE MRI

**DOI:** 10.1002/nbm.70299

**Published:** 2026-05-07

**Authors:** Céline Smekens, Pieter Van Dyck, Patrick S. Fuchs, Jan Sijbers, Thomas Janssens, Ben Jeurissen

**Affiliations:** ^1^ imec‐Vision Lab, Department of Physics University of Antwerp, Universiteitsplein 1 Antwerp Belgium; ^2^ Siemens Healthcare NV/SA, Alfons Gossetlaan 54 Dilbeek Belgium; ^3^ Department of Radiology Antwerp University Hospital, Drie Eikenstraat 655 Antwerp Belgium; ^4^ MIRA, Faculty of Medicine and Health Sciences University of Antwerp, Universiteitsplein 1 Antwerp Belgium; ^5^ μNEURO Research Centre of Excellence University of Antwerp, Universiteitsplein 1 Antwerp Belgium

**Keywords:** data‐driven decomposition, knee joint, non‐negative matrix factorization, quantitative MRI, tissue characterization, ultrashort echo time

## Abstract

T_2_* quantification based on multi‐echo ultrashort echo time (UTE) MRI has been widely proposed for knee tissue characterization, yet accurate model‐based parameter estimation remains challenging due to ill‐conditioning of the corresponding inverse problems. Alternatively, data‐driven methods such as non‐negative matrix factorization (NMF) can derive tissue features without relying on biophysical model assumptions. This study aimed to investigate the use of an NMF‐based framework informed by 3D multi‐echo UTE MRI for multi‐compartmental analysis of healthy and impaired knee structures. To this end, six asymptomatic adults and 13 patients underwent 3‐T MRI, including routine anatomical sequences, and a 3D UTE spiral research sequence with six echo times. Convexity‐constrained NMF was used to identify characteristic signal components from UTE T_2_*‐weighted datasets. Basis functions were determined based on asymptomatic knee data and used to compute subject‐specific weight maps. Normalized weight distributions were computed for 13 regions of interest across asymptomatic knees and contrasted with median weights of selected structures in patients. The application of the NMF‐based framework yielded four reproducible basis functions associated with fast decay, slow decay, water–fat mixing, and fat‐like behavior. Knee structures with known short T_2_* showed a dominant fast‐decaying component (median weights ≥ 0.68), while structures with long T_2_* showed a greater contribution from the slowly‐decaying component (median weights ≥ 0.44). The fat‐like component was predominant in Hoffa's fat pad, while the water–fat mixture‐related component's contribution was overall low. Lesioned menisci and ligaments generally displayed reduced fast‐decaying and increased slowly decaying component contributions. These findings suggest that convexity‐constrained NMF of 3D multi‐echo UTE MRI is feasible for data‐driven knee tissue characterization as it extracts biophysically related signal components and describes their relative contribution to the measured signal in various knee tissues. Moreover, the proposed framework shows potential for differentiation between asymptomatic and impaired knee tissues, particularly in the posterior horn of the medial meniscus and the anterior cruciate ligament, and consequently holds promise for more objective diagnosis and monitoring of internal knee derangements.

## Graphical abstract

Non‐negative matrix factorization (NMF) can derive tissue features in a data‐driven manner without relying on biophysical model assumptions. First‐time application of NMF to 3D multi‐echo UTE knee MRI revealed four reproducible components associated with tissues of fast and slow transverse relaxation, water–fat mixture (chemical shift), and fat tissue. Overall, impaired menisci and ligaments exhibited a clear shift in composition compared to healthy controls, and consequently, the proposed data‐driven method may help identify quantitative imaging biomarkers of tissue disease and degeneration.

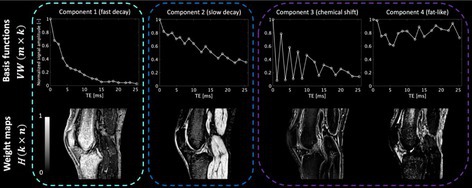

**Abbreviations**
ACL: anterior cruciate ligament
aLM: anterior horn of lateral meniscus
BMI: body mass index
BSS: blind source separation
CAIPIRINHA: controlled aliasing in parallel imaging results in higher acceleration
CRLB: Cramér–Rao lower bound
DDSS: dual‐density spiral sampling
DRGS: Deep Resolve Gain and Sharp
eBone: femoral bone (epiphysis)
FA: flip angle
FatSat: spectral fat saturation
FOV: field of view
FS: fat‐suppressed
GM: gastrocnemius muscle
GRAPPA: generalized autocalibrating partially parallel acquisitions
GRE: gradient echo
HFP: Hoffa's fat pad
IQR: interquartile range
IW: intermediate‐weighted
LDR: linear dimensionality reduction
MAE: magic angle effect
mBone: femoral bone (metaphysis)
ME: multi‐echo
MRI: magnetic resonance imaging
NMF: non‐negative matrix factorization
PC: patellar cartilage
PCL: posterior cruciate ligament
pLM: posterior horn of lateral meniscus
pMM: posterior horn of medial meniscus
PT: patellar tendon
qMRI: quantitative magnetic resonance imaging
ROI: region of interest
SD: standard deviation
SM: semimembranosus muscle
SPACE: sampling perfection with application optimized contrast using different flip angle evolutions
SPAIR: spectral attenuated inversion recovery
SPIRiT: iterative self‐consistent parallel imaging reconstruction
tBone: tibial bone
TE: echo time
TR: repetition time
TSE: turbo spin echo
UTE: ultrashort echo time
VIBE: volumetric interpolated breath‐hold examination

## 1 Introduction

Conventional multi‐contrast turbo spin echo (TSE) MRI followed by qualitative analyses remains the modus operandi of diagnostic knee MRI to date [1, 2]. Yet, the rapid and widespread increase of biophysics‐based and artificial intelligence‐driven quantitative techniques is gaining clinical interest in the context of precision medicine [3, 4]. Quantitative MRI (qMRI) enables the identification of quantitative imaging biomarkers to assess the biochemical composition and (micro)structural integrity of tissues, allowing for objective comparisons over time, across patients, and between study protocols, systems, and sites [3, 5]. Consequently, qMRI holds promise to reveal early structural degeneration, enhance lesion differentiation, and monitor treatment response [5].

Musculoskeletal qMRI data acquisition benefits from the use of ultrashort echo time (UTE) sequences as these pulse sequences are particularly valuable for capturing tissues with fast transverse relaxation (T_2_/T_2_*), which display very low signal intensity on conventional MRI [6, 7]. As knee tissues exhibit a mix of super short (< 0.1 ms), ultrashort (0.1–1.0 ms), short (1–10 ms), and long (> 10 ms) T_2_/T_2_* relaxation components related to distinct proton pools [8], UTE T_2_* quantification has been widely proposed to characterize tendons, ligaments, menisci, articular cartilage, and bone [8, 9, 10, 11, 12].

Quantitative UTE T_2_* datasets are generally acquired with gradient echo (GRE)‐based sequences using short excitation pulses and efficient non‐Cartesian k‐space encoding trajectories (e.g., radial, spiral, and cones) to capture rapidly relaxing tissue signals [13]. The optimal echo time (TE) range and distribution depend on several factors including the required tissue sensitivity, the chosen analysis, and the available scan time [9, 14].

To estimate T_2_* values, mono‐ and bi‐exponential T_2_* relaxation models are commonly employed [15, 16, 17]. The popular mono‐exponential model is relatively simple but lacks specificity [3], whereas the bi‐exponential model incorporates specificity through the identification of a short and a long T_2_* component. More complex models, including stretched‐exponential and multi‐exponential approaches, offer additional insights and have previously been applied to UTE knee MRI [18, 19, 20]. However, these models usually result in ill‐conditioned inverse problems, where small perturbations of the measured signal (e.g., due to noise) can lead to large variations in the estimated parameters, thereby posing challenges to conventional model fitting approaches and potentially leading to unreliable parameter estimation [21].

Alternatively, data‐driven approaches such as blind source separation (BSS) techniques, which omit underlying assumptions of biophysical models and use a minimum of prior information can be employed for tissue characterization [22]. A widely adopted BSS subcategory consists of unsupervised linear dimensionality reduction (LDR) techniques. These techniques analyze all measurements simultaneously and provide a low‐rank approximation, i.e., an approximate factorization, for the data matrix at hand. The various LDR methods primarily differ in the constraints they impose on the matrix factors: e.g., singular value decomposition and principal component analysis enforce orthogonality of the singular vectors and principal components, respectively, whereas independent component analysis seeks statistically independent components [23]. While these methods can provide low‐error approximations, their components may be more difficult to interpret as they allow mixtures of positive and negative contributions [24]. In contrast, non‐negative matrix factorization (NMF) imposes non‐negativity constraints on the matrix factors and provides in this way a non‐negative, parts‐based representation [24]. For naturally non‐negative magnitude MRI data, NMF may thus offer a more easily interpretable LDR alternative. More specifically, NMF approximates non‐negative input data by a product of two non‐negative low‐rank matrices: a basis matrix representing fundamental features and a weight matrix indicating their weighted spatial distribution [23, 25]. The resulting parts‐based representation allows for more easily interpretable and potentially physically meaningful components across a wide range of applications [23].

In MRI, NMF has been proposed for, among others, brain multi‐tissue decomposition based on diffusion‐weighted MRI [25, 26], myelin water fraction mapping [27], and tumor characterization [28]. Relevantly, Christiaens et al. imposed a convexity constraint to limit the basis functions to be convex combinations of the individual voxel signals, enhancing physical plausibility [25].

Given the potential of multi‐echo UTE MRI for knee tissue characterization, the challenges of model‐based parameter estimation, and the capability of NMF to derive fundamental signal features in a data‐driven manner, this study aimed to explore the use of convexity‐constrained NMF for multi‐component analysis of 3D multi‐echo UTE knee MRI in asymptomatic volunteers and patients.

## 2 Methods

### 2.1 Study Design

The institutional ethics committee approved this prospective study (registration number B300201627688), which was conducted in compliance with the Declaration of Helsinki. Written informed consent was obtained from all subjects prior to participation.

Six asymptomatic volunteers with no knee pain/discomfort and no known/suspected knee pathology (i.e., healthy controls), and 14 patient volunteers with clinical indications for knee MRI were recruited into the main study. One patient was excluded as high‐grade chronic degenerative osteoarthritis was observed, jeopardizing the segmentation of structures of interest. The demographics and clinical characteristics of the subjects enrolled in the main study are listed in Table 1.

**TABLE 1:** Demographics and clinical characteristics of the asymptomatic volunteers and patients enrolled in the main study.

Characteristic		Asymptomatic volunteers	Patients
Sex, *n*	Female	3	3
	Male	3	10
Age, years, mean ± SD (range)	All	30.3 ± 6.1 (23–39)	43.0 ± 15.1 (22–72)
BMI, kg/m^2^, mean ± SD (range)	All	22.8 ± 1.9 (20.9–25.5)	26.2 ± 4.2 (20.6–35.2)
Laterality, *n*	Left	2	6
	Right	4	7
Clinical indication for MRI, *n*	Acute trauma	—	3
	Chronic pain	—	10

*Note: n*, number.

**FIGURE 1:**
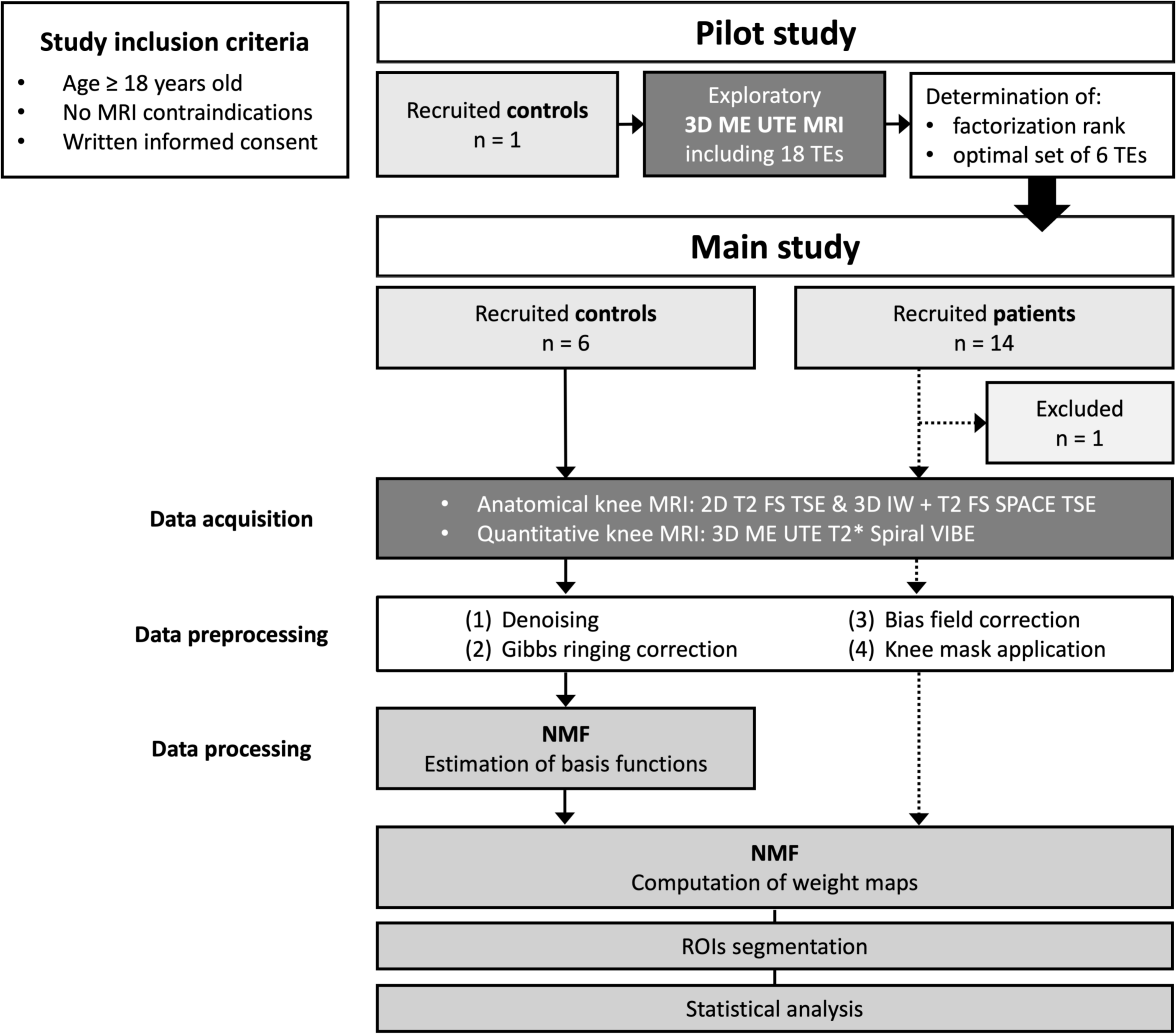
Diagram of the study design.

Prior to the main study, a pilot study was conducted in which one asymptomatic volunteer (male, right knee, 39 years, BMI: 25.5 kg/m^2^) underwent an extended examination to inform the main study's research protocol and the postprocessing of the quantitative data. A diagram of the study design is shown in Figure 1.

### 2.2 MRI Acquisition

All subjects were scanned on a 3‐T MRI scanner (MAGNETOM Vida XT, Siemens Healthineers, Forchheim, Germany) using an 18‐channel transmit/receive knee coil.

The protocol included a 2D T_2_‐weighted fat‐suppressed (FS) TSE sequence and 3D intermediate‐weighted (IW) and T_2_‐weighted FS CAIPIRINHA SPACE TSE sequences [29]. In addition, all subjects were imaged with a research application enabling UTE MRI. The 3D UTE spiral VIBE sequence was used to acquire T_2_*‐weighted datasets based on a stack‐of‐spirals k‐space trajectory with adaptive TE, in‐plane acceleration using dual‐density spiral waveforms, and in‐line reconstruction with an iterative self‐consistent parallel imaging reconstruction (SPIRiT)‐based algorithm [30, 31].

For every subject, 6 T_2_*‐weighted volumes were acquired at an optimized set of TEs with a field‐of‐view of 180 × 180 × 142 mm^3^ and a voxel size of 0.8 × 0.8 × 0.8 mm^3^ in a scan time of 10:13 min. The total acquisition time of the main study protocol was 21:38 min. The main imaging parameters are provided in Table 2.

**TABLE 2:** Imaging parameters of the 2D TSE, 3D SPACE TSE and 3D UTE spiral VIBE sequences.

Parameter	2D TSE	3D SPACE TSE		3D UTE spiral VIBE
Contrast	T_2_ FS	IW	T_2_ FS	T_2_*
Orientation	Sagittal	Sagittal	Sagittal	Sagittal
Fat suppression	FatSat	—	SPAIR	—
Acceleration technique	GRAPPA + DRGS	CAIPIRINHA	CAIPIRINHA	DDSS + SPIRiT
Acceleration factor	2	4	4	2
Echo train length	11	60	44	—
Receiver bandwidth (Hz/pixel)	252	422	416	—
Number of slices	39	240	192	178
Acquisition matrix (freq × phase)	336 × 252	320 × 320	256 × 256	224 × 224
FOV (mm^2^)	140 × 140	160 × 160	160 × 160	180 × 180
Voxel size (mm^3^)	0.2 × 0.2 × 2.5	0.5 × 0.5 × 0.5	0.6 × 0.6 × 0.6	0.8 × 0.8 × 0.8
TR (ms)	4530	900	1000	28
TE (ms)	64	28	108	0.06, 4.92, 8.61, 12.30, 20.00, 23.37
FA (°)	180	120	Variable	6
Spiral interleaves (number)	—	—	—	246
Spiral readout duration (μs)	—	—	—	1160
Total scan time (min:s)	2:54	3:52	4:39	10:13

In the pilot study, the imaging protocol consisted of three repeated acquisitions of the 3D UTE spiral VIBE sequence. Parameters were set as listed in Table 2, except for the repetition time (TR), which was adjusted to 30 ms to accommodate different sets of TEs across repetitions: (1) “in‐phase” TEs: 2.46, 4.92, 7.38, 12.30, 17.22, 22.14 ms; (2) “out‐of‐phase” TEs: 1.23, 3.69, 8.61, 13.53, 18.45, 23.37 ms; (3) “intermediate” TEs: 0.06, 6.00, 10.00, 15.00, 20.00, 25.37 ms. The different sets of TEs were included to enable capturing a broad range of signal evolutions. The “in‐phase” and “out‐of‐phase” TEs were based on the difference in resonance frequency between water and fat protons at 3‐T. The total acquisition time of the extended research protocol was 35:24 min.

### 2.3 Data Processing

#### 2.3.1 Data Preprocessing

First, the complex‐valued T_2_*‐weighted images were denoised using random matrix theory [32, 33]. Secondly, the magnitude images of the denoised complex images were corrected for Gibbs ringing artifacts [33, 34]. Next, N4 bias field correction was performed, with the bias field computed from the first echo image using the same parameter settings across subjects [35, 36]. Finally, a conservative full knee mask was defined on the first echo image through manual thresholding and successive morphological operations (i.e., dilation and erosion). In the pilot study, all T_2_*‐weighted images additionally underwent rigid registration to the shortest TE image prior to the bias field correction [36].

#### 2.3.2 NMF

The problem of identifying multiple characteristic signal components from a set of preprocessed 3D UTE T_2_*‐weighted images was formulated as a convexity‐constrained NMF problem [37]:

$$\min_{W,H}{\left\|V-\mathrm{VWH}\right\|}_{2}^{2}\;\text{subject to}\;W=\left[w_{\mathrm{ij}}\right]\geq 0,H=\left[h_{\mathrm{ij}}\right]\geq 0,\sum_{i=1}^{n}w_{i,j}=1$$

**FIGURE 2:**
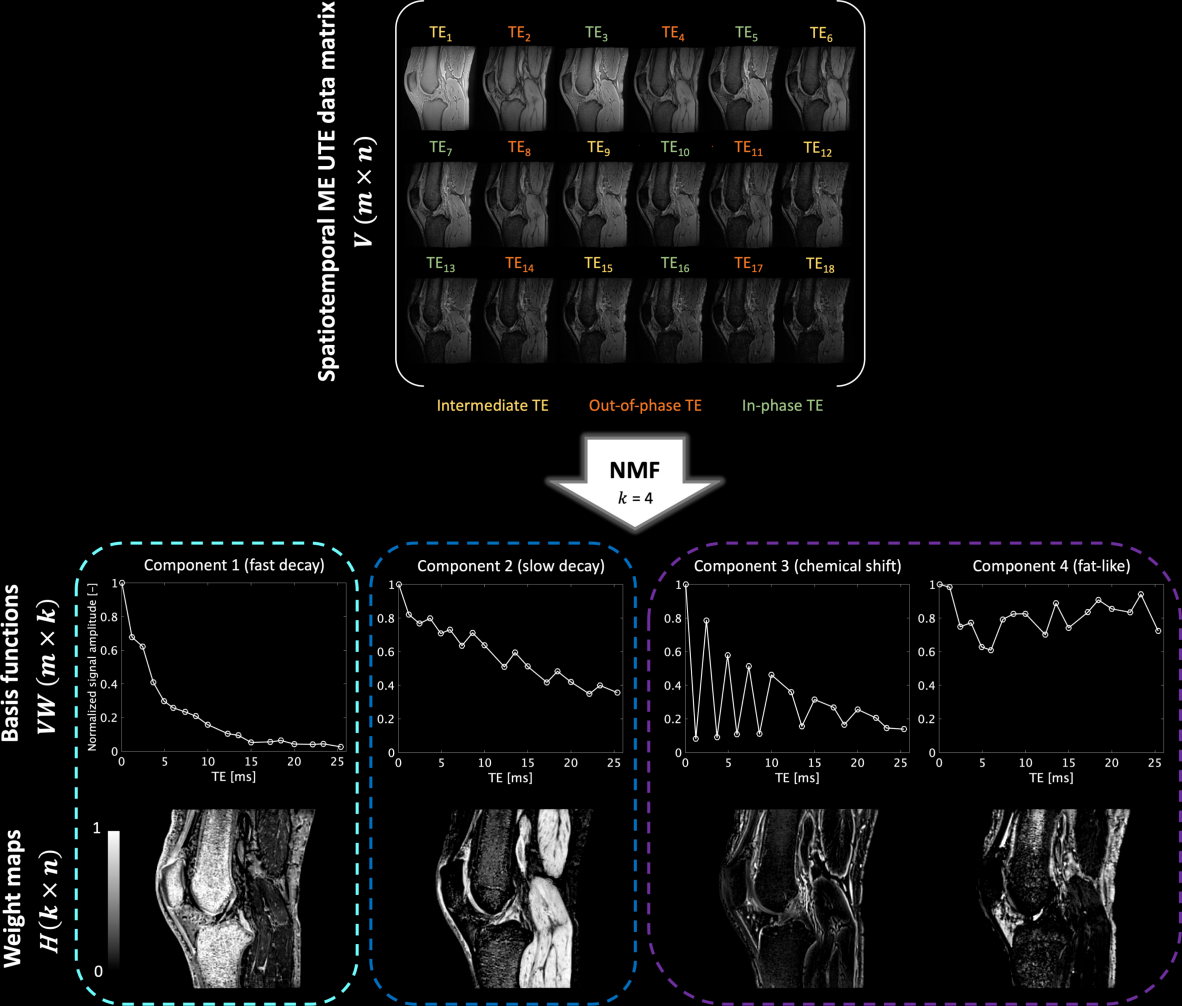
Illustration of NMF applied to the 3D multi‐echo UTE knee data from the asymptomatic volunteer in the pilot study. The spatiotemporal matrix V (m‐by‐n) comprises the preprocessed 3D UTE T_2_*‐weighted images acquired at 18 different TEs (i.e., 6 in‐phase, 6 out‐of‐phase and 6 intermediate TEs). m represents the number of TEs and n the number of voxels in the 3D knee volume. k is the factorization rank. Resulting from the NMF, VW(m‐by‐k) contains the set of k components or non‐negative basis functions, and H (k‐by‐n) the associated non‐negative weight maps. The shown basis functions are normalized to the maximal signal intensity (at TE_1_) and the weight maps are normalized to sum up to 1.

The spatiotemporal data matrix $V\in {\mathbb{R}}_{+}^{m\times n}$ represents the non‐negative 3D UTE T_2_*‐weighted measurements, where each row up to m corresponds to a different measurement at a particular TE and each column up to n to a different voxel in the 3D volume. The matrix $\mathrm{VW}\in {\mathbb{R}}_{+}^{m\times k}$ is defined as the set of k non‐negative basis functions, each of which is a convex combination of the columns of V. The matrix $W\in {\mathbb{R}}_{+}^{n\times k}$ thus provides the weight of each data vector for each basis function, and $H\in {\mathbb{R}}_{+}^{k\times n}$, the associated non‐negative weight matrix, specifies the weights for the linear combination of basis functions to reconstruct each data vector. In this low‐rank approximation, k is the factorization rank and thus the number of characteristic tissue components. A schematic representation of NMF applied to multi‐echo UTE knee data from the pilot study is shown in Figure 2.

Factorizations were performed in MATLAB (R2020a, The Mathworks Inc., Natick, MA; United States). To solve the convexity‐constrained NMF problem, W was initialized with *K*‐means clustering and H as described in Ding et al. [37]. Subsequently, W and H were alternately (re)estimated under the non‐negativity and convexity constraints. Convergence of the iterative decomposition was set to a maximum of 100,000 iterations or a relative change in the residual error between consecutive iterations of less than 10^−5^. Convergence was consistently achieved well before reaching the maximum number of iterations. The employed NMF algorithm is available at https://github.com/audiofilter/nmflib.

#### 2.3.3 Factorization Rank and Optimal TEs

The factorization rank k and the optimal set of TEs were determined from the NMF analysis of the pilot dataset. A sparse mask comprising ~9000 voxels randomly distributed across the 3D knee mask was used to determine the input for the NMF. In search of the optimal k value, the NMF was repeated for all values of k ranging between 2 and 6 (i.e., the maximum number of measurements in a single UTE acquisition). The residual error at convergence and the visual assessment of the resulting k weight maps informed by prior knowledge of the anatomy and T_2_* relaxation behavior of knee structures, guided the rank determination [22, 23, 25].

To determine the optimal selection of six TEs for the 3D multi‐echo UTE data acquisition, the Cramér–Rao Lower Bounds (CRLBs) on the variances of the non‐negative basis functions' coefficients were used as described in a previous study [38]. The Fisher information matrix was calculated for all possible combinations of six out of 18 TEs, which obeyed the sequence‐imposed minimal echo spacing constraint of 2.31 ms. The subset of six TEs resulting in the lowest sum of CRLBs was deemed optimal and used in the main study.

#### 2.3.4 General Basis Functions

To extract signal components informative of healthy knee tissues, the UTE datasets of the six asymptomatic volunteers were used. First, signal intensity histograms of all asymptomatic datasets were matched to the histogram of an arbitrarily chosen asymptomatic reference dataset to harmonize inter‐scan variability of the signal intensity [39]. Subsequently, randomly generated sparse masks (one per volunteer, each comprising an average of ~8500 voxels) were applied to the datasets. These samples were then aggregated into a composite matrix of normalized asymptomatic data. In a joint decomposition, NMF was applied to this matrix to yield k general basis functions, which were used to calculate corresponding weight maps for both asymptomatic volunteers and patients.

#### 2.3.5 Reproducibility Analysis

The intra‐subject reproducibility of the decomposition into k signal components was evaluated for the extensive asymptomatic dataset from the pilot study (including 18 TEs) by repeating the NMF for three different sparse masks of equal size (i.e., ~9000 voxels) randomly sampled from the 3D knee mask and comparing the resulting basis functions.

The inter‐subject reproducibility of the decomposition was evaluated by separately applying the NMF to each of the six individual asymptomatic datasets of the main study (including six TEs). The median and corresponding ranges of the resulting basis functions were compared to the general basis functions.

#### 2.3.6 Weight Maps

UTE datasets of all subjects in the main study were histogram‐matched to the same asymptomatic reference, as previously described. Given the predetermined matrix of general basis functions, a weight matrix was computed for every subject by solving a convex non‐negative least‐squares problem for all knee voxels [23].

To facilitate the quantitative analysis, the compartment weights were normalized to sum to 1 in every voxel. Furthermore, regions of interest (ROIs) were manually drawn on the 3D SPACE TSE images after rigid registration to the first echo image of the UTE dataset [36]. In asymptomatic datasets, ROIs were manually delineated in the anterior cruciate ligament (ACL), posterior cruciate ligament (PCL), patellar tendon (PT), anterior horn of lateral meniscus (aLM), posterior horn of lateral meniscus (pLM), posterior horn of medial meniscus (pMM), gastrocnemius muscle (GM, medial head), semimembranosus muscle (SM), patellar cartilage (PC), Hoffa's fat pad (HFP), femoral bone (eBone, epiphysis; mBone, metaphysis), and tibial bone (tBone). In patient datasets, ROIs were drawn in the aLM, pLM, pMM, ACL, and eBone, and in structures showing abnormalities. The considered knee derangements included meniscal tears, tears in ligaments, tendons and muscles (fiber discontinuity greater than 50%), cartilage defects (Grade 3 and 4, Outerbridge Classification), and bone injuries (including edema and fractures) [29]. ROIs were verified by a musculoskeletal radiologist (P.V.D.) with 21 years of experience.

### 2.4 Statistical Analysis

The descriptive statistical analysis was performed in R (v4.2.3, R Foundation for Statistical Computing, Vienna, Austria).

For asymptomatic volunteers, the median and interquartile range (IQR) of the median normalized compartment weights were computed for all compartments and ROIs. For the aLM, pLM, pMM, ACL, and eBone, this information was additionally contrasted with the medians of normalized compartment weights computed for patients.

## 3 Results

### 3.1 Pilot Study

**FIGURE 3:**
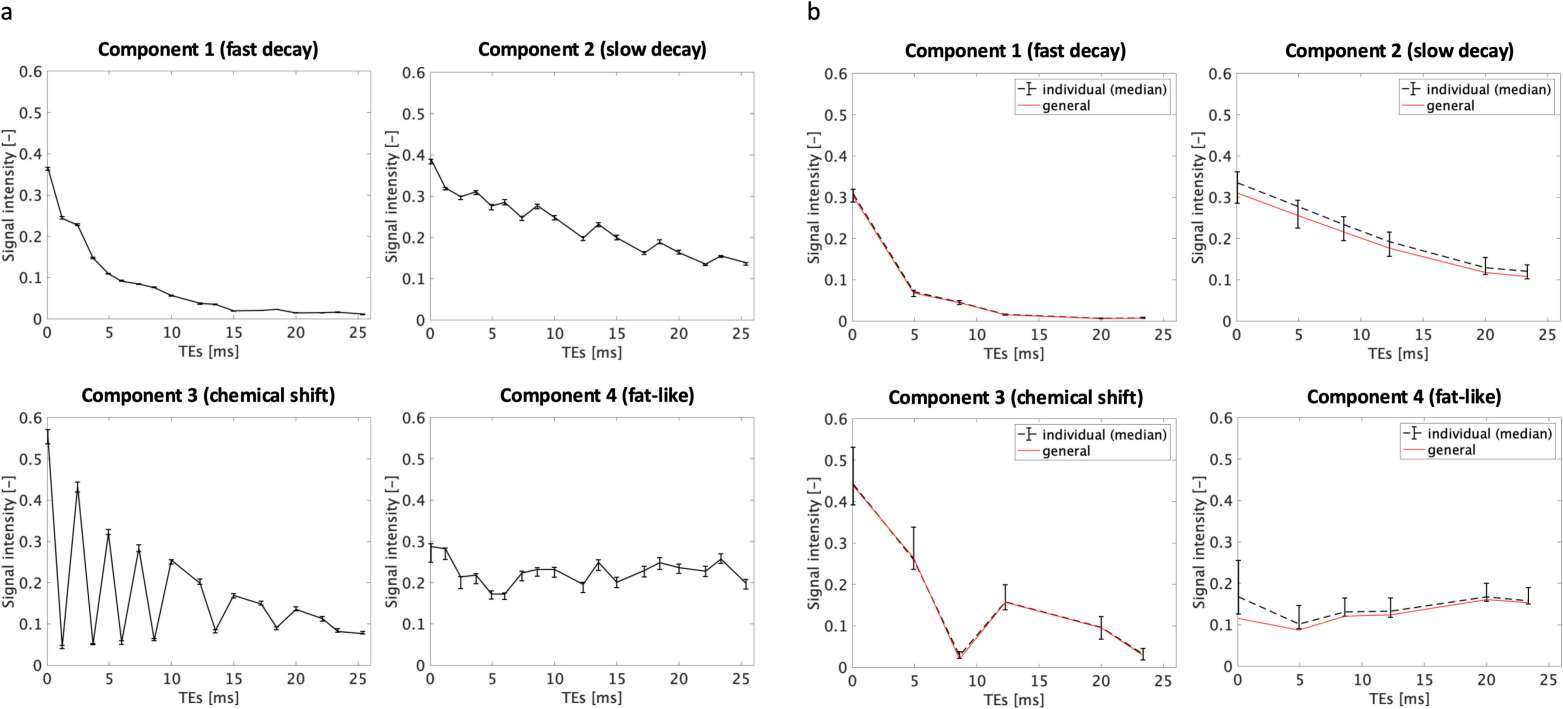
Intra‐subject (a) and inter‐subject (b) reproducibility of the four characteristic signal components (i.e., basis functions). The median values and ranges (i.e., error bars for minimum and maximum values) of the basis functions are shown for each component. Additionally, the general basis functions resulting from the NMF of the aggregated asymptomatic data are displayed (b, solid red). Overall, the small signal variations in (a) indicate good intra‐subject reproducibility. In (b), the general basis functions lie within the ranges of the individually computed basis functions, except for the general fat‐like component at the first echo time. The fast‐decaying component shows excellent inter‐subject reproducibility.

In the pilot study, the optimal rank k for the convexity‐constrained NMF of UTE knee MRI was found to be 4 as this resulted in the best visual discrimination of anatomical structures. The input data was decomposed into four basis functions (Figure 2) of which two resemble characteristic exponentially decaying T_2_* signals (component 1: fast decay/short T_2_*, component 2: slow decay/long T_2_*), and the remaining two display oscillating behavior relatable to signal from a water–fat mixture (component 3, alternatively referred to as “chemical shift”) and a fat‐like signal (component 4). The decomposition exhibited good intra‐subject reproducibility as only small signal variations were observed for the repeated NMFs using different sparse masks, as shown in Figure 3a.

The CRLB analysis further indicated the optimal set of six TEs for the UTE data acquisition in the main study (Table 2). The selected TEs are spaced over the considered range and include the shortest acquirable TE for the given sequence settings, 2 in‐phase TEs, 2 out‐of‐phase TEs, and the second‐longest intermediate TE.

Using the predetermined rank k=4, the NMF of the merged asymptomatic datasets in the main study gave rise to four general basis functions as depicted in Figure 3b. These general basis functions display the same characteristic signal behaviors as in the pilot study. Moreover, applying the NMF separately to the individual asymptomatic datasets resulted in good inter‐subject reproducibility, especially for the fast‐decaying component, which showed very small variations of the basis function across the repeated NMFs (Figure 3b). All general basis functions were found to lie within the ranges of the individually computed basis functions, except for the general fat‐related signal which showed a deviation (i.e., a lower value outside of the range) at the first echo time.

### 3.2 Compartment Weights for Asymptomatic Volunteers

**FIGURE 4:**
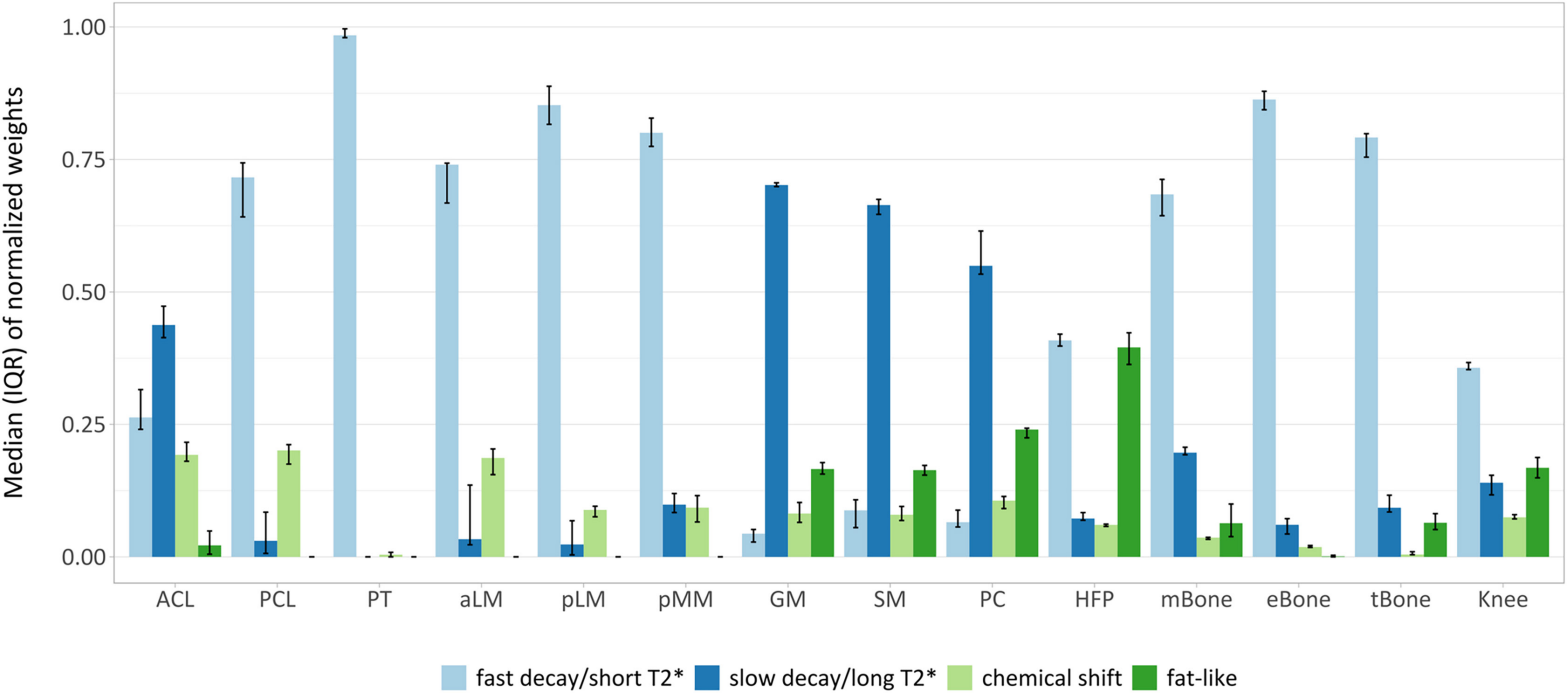
Medians and interquartile ranges (IQRs) of the normalized compartment weights of asymptomatic volunteers. Regions of interest (ROIs) were delineated in the full knee (Knee) and in 13 knee structures. The median and the IQR (shown as error bars) of the volunteers' medians of normalized weights were computed for every compartment (from left to right: light blue, fast decay/short T_2_*; dark blue, slow decay/long T_2_*; light green, water–fat mixture‐related (chemical shift); dark green, fat‐like) and every ROI. Note that knee structures with known short mean T_2_* relaxation display a dominant contribution of the fast‐decaying signal component (PCL, PT, aLM, pLM, pMM, mBone, eBone, and tBone), while structures with longer mean T_2_* relaxation show greater contributions of the slowly‐decaying component (ACL, GM, SM, and PC). The fat‐like compartment shows the highest contribution in the HFP ROI. The component related to water–fat mixing (chemical shift) makes up a relatively low portion of the signal in all ROIs.

Figure 4 presents bar charts of the normalized weights reflecting the relative presence of characteristic signal compartments in the UTE input data of asymptomatic volunteers. For every ROI, the median and IQR of the median normalized weights are displayed by compartment. Knee structures with known short mean T_2_* relaxation times, i.e., PCL, PT, aLM, pLM, pMM, mBone, eBone, and tBone, showed a dominant contribution of the fast‐decaying signal component (median normalized weights between 0.68 and 0.98). Knee structures with demonstrated long T_2_* relaxation behavior, i.e., ACL, GM, SM, and PC, showed greater contributions of the slowly decaying basis function (median normalized weights between 0.44 and 0.70). The ACL also showed an important contribution of the fast‐decaying signal (median normalized weight of 0.26). The HFP demonstrated relatively higher and nearly equal weights for the short T_2_* and fat‐like compartments (median normalized weights of 0.41 and 0.40, respectively). Moreover, the fat‐like compartment showed the second highest contribution in GM, SM, and PC (median normalized weights between 0.16 and 0.24) and was marginally present in ACL, mBone, and tBone, while it did not contribute to the remaining structures. The compartment related to water–fat mixing (chemical shift) made up a relatively low portion of the signal in all structures (below 0.20 for ACL, PCL, aLM, and PC, and below 0.10 for other ROIs). Furthermore, the IQRs (shown as error bars in Figure 4) were overall relatively narrow with some exceptions including the slowly decaying compartments of PCL, aLM, and pLM and the fat‐like compartment of ACL and mBone. Finally, the weight distribution for the asymptomatic knee considered in its entirety showed a prevalence of the fast‐decaying component, secondary contributions of the slowly‐decaying and fat‐like compartments accompanied by relatively larger IQRs, and a minor contribution of the water–fat mixture‐related signal (median normalized weights of 0.36, 0.14, 0.17, and 0.07, respectively).

### 3.3 Compartment Weights for Pathological Knee Structures

Structural abnormalities meeting the study's lesion inclusion criteria included 14 meniscus lesions (aLM, *n* = 3; pLM, *n* = 6; pMM, *n* = 5), 4 ACL lesions, 7 cartilage lesions (medial femoral condyle, *n* = 2; lateral femoral condyle, *n* = 1; medial tibial plateau, *n* = 2; patella, *n* = 2), and 7 bone injuries (femur, *n* = 4; tibia, *n* = 3). The analysis of normalized compartment weights for pathological knee structures was focused on pMM, aLM, pLM, ACL, and eBone.

**FIGURE 5:**
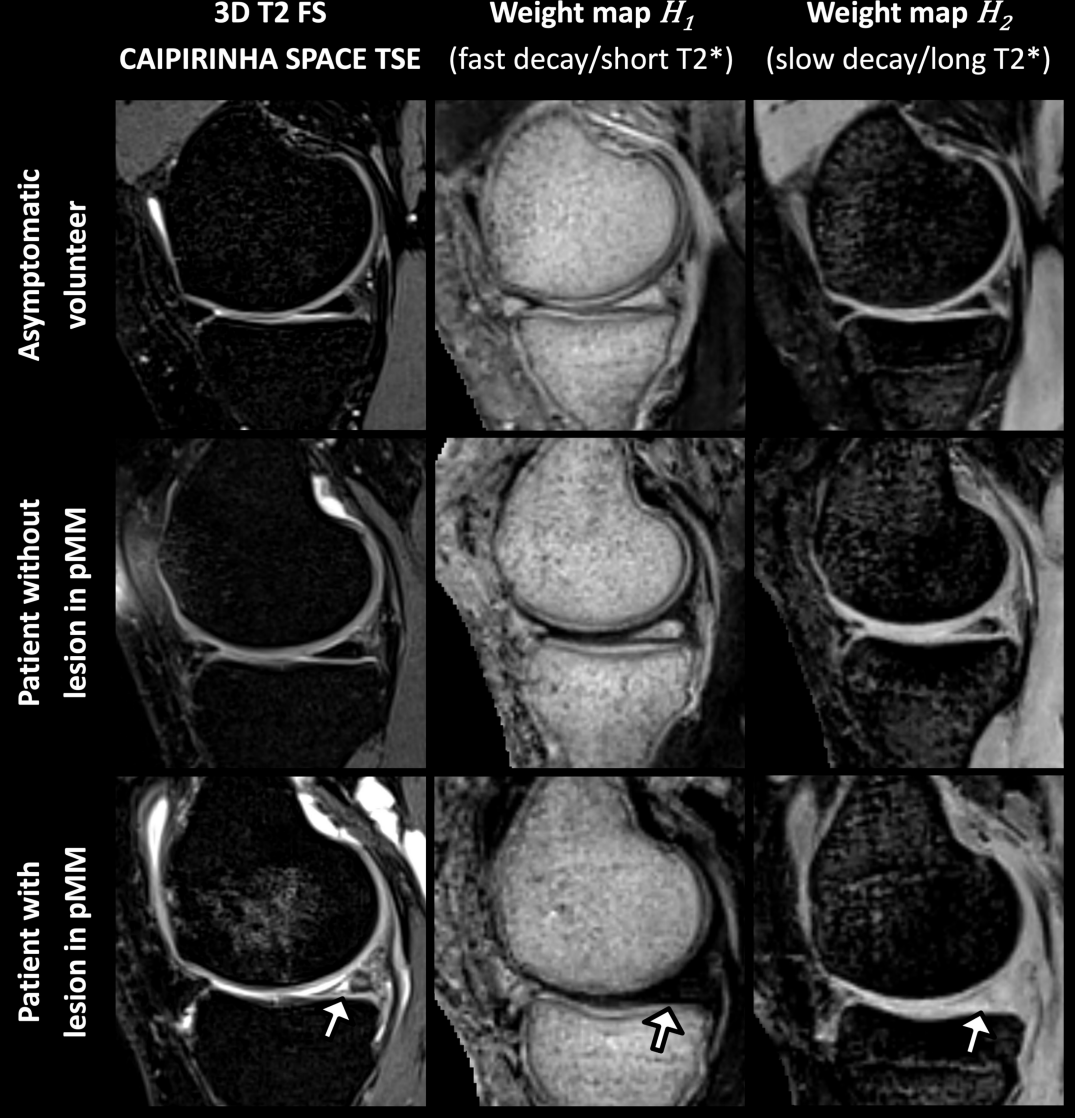
Sagittal 3D T_2_ FS CAIPIRINHA SPACE TSE images and weight maps $H_{1}$ and $H_{2}$, respectively corresponding to the fast‐ and slowly‐decaying characteristic signal components, of the left knee of a 23‐year‐old female asymptomatic volunteer (top row), the right knee of a 33‐year‐old female patient with sustained knee pain (middle row) and the left knee of a 72‐year‐old female patient reporting episodes of knee giving away (bottom row). No lesions were observed in the posterior horn of the medial meniscus (pMM) of the 33‐year‐old female patient on anatomical MR images, while a complex tear was noted in the pMM of the 72‐year‐old female patient (white arrows). The lesioned pMM showed lower weights (darkening) in the weight map $H_{1}$ and increased weights (brightening) in the weight map $H_{2}$ in comparison to the maps of the asymptomatic volunteer. For the female patient with no detected pMM lesion, a slight darkening in $H_{1}$ and shallow brightening of $H_{2}$ can be observed for the pMM region. Weight maps for the fat‐like component and the component related to the water–fat mixing (chemical shift) are not shown as the contributions of these components in the pMM region were negligible.

**FIGURE 6:**
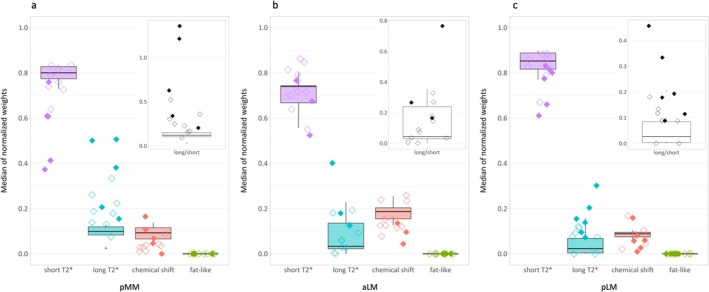
Median normalized compartment weights for the menisci of asymptomatic volunteers and patients. Three meniscal regions are considered: (a) posterior horn of medial meniscus (pMM), (b) anterior horn of lateral meniscus (aLM), and (c) posterior horn of lateral meniscus (pLM). Box and Whisker Plots represent the distribution of the median weights of the 6 asymptomatic volunteers per compartment (from left to right: purple, fast decay/short T_2_*; blue, slow decay/long T_2_*; red, water–fat mixture‐related (chemical shift); green, fat‐like). The medians of normalized weights computed for patients with meniscus lesions are presented individually (filled diamonds) next to the medians for patients with injuries solely outside of the considered meniscus region of interest (empty diamonds). Similar data visualization is used in the top right graphs which represent ratios of long T_2_* to short T_2_* median normalized compartment weights. Overall, lesioned menisci display a trend toward lower weights for the short T_2_*‐like signal component and higher weights for the long T_2_*‐like component, which is highlighted by increased weight ratios for the patients (especially for the pMM and pLM ROIs).

Figure 5 provides an example of the weight maps corresponding to the fast‐ and slowly‐decaying signal compartments for an asymptomatic volunteer (top row), a patient with no pMM lesion (middle row) and a patient with a complex tear in the pMM (bottom row). Median normalized weights computed for the pMM of asymptomatic volunteers and patients with and without pMM lesions are displayed in Figure 6a. Box and Whisker Plots represent the distribution of median weights for the six asymptomatic datasets. The weights computed for the five patients with pMM lesions are presented individually (filled diamonds) and fall outside of the asymptomatic IQRs for the short and long T_2_* compartments. More specifically, lesioned pMMs generally showed a shift towards lower contributions of the fast‐decaying component and increased contributions of the long T_2_* component. A similar yet less pronounced trend was observed for patients with no pMM lesions but other knee derangements (empty diamonds). For all subjects, the fat‐like compartment was not present in pMM and the water–fat mixture‐related compartment (chemical shift) showed a similar or reduced contribution for patients compared with asymptomatic pMMs. The observations made for pMM weights also generally apply to aLM and pLM weights but deviations from the asymptomatic data were less pronounced (Figure 6b,c, respectively). The differences between asymptomatic and patient compartment weights were enhanced by ratios of long to short T_2_* compartment weights (top right subplots in Figure 6). The weight ratios of patients with meniscus lesions were generally higher than the ratios of asymptomatic volunteers, except for aLMs, which demonstrated wider distributions of the short and long T_2_* compartment weights for the asymptomatic volunteers.

**FIGURE 7:**
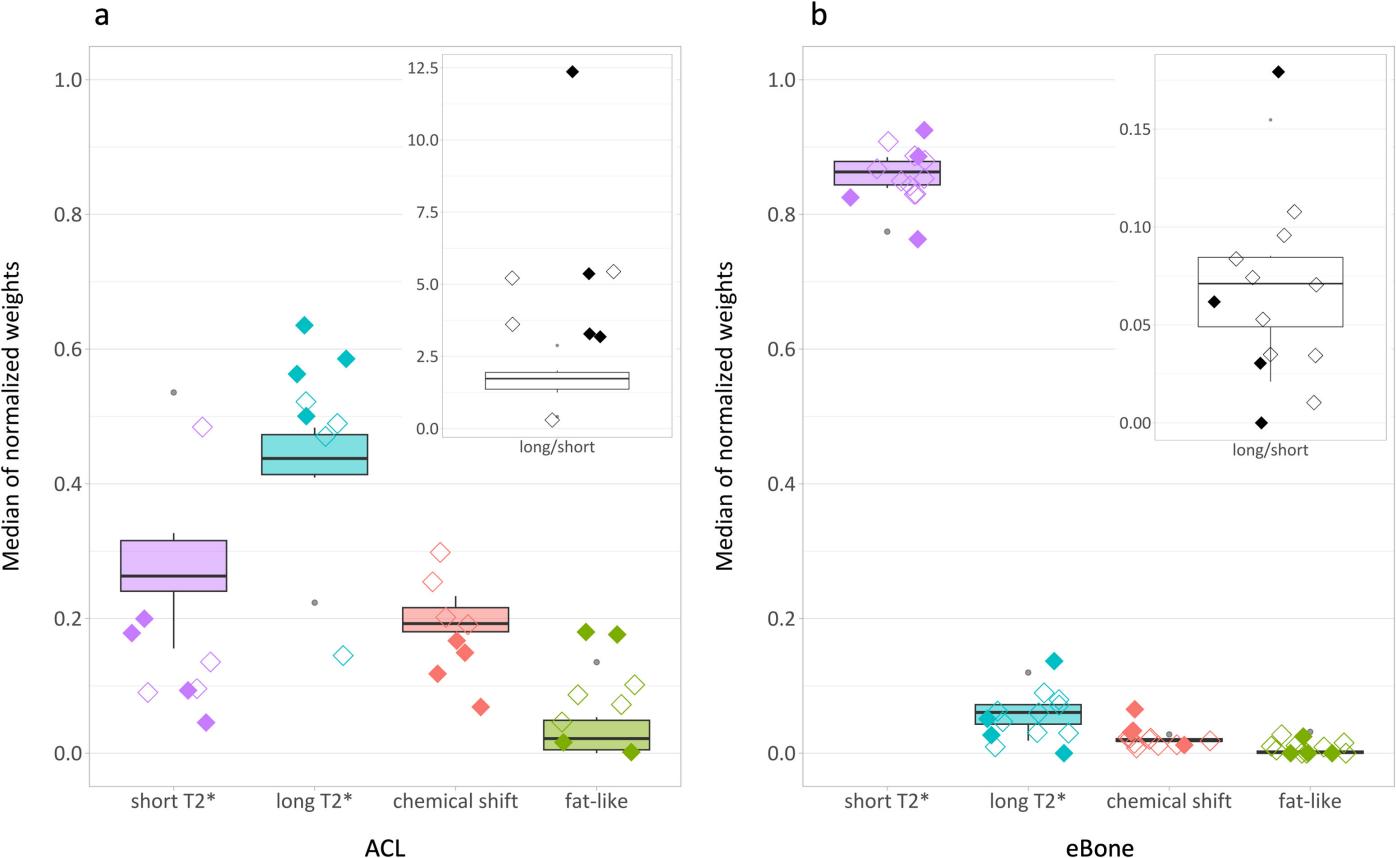
Median normalized compartment weights of asymptomatic volunteers and patients for the (a) anterior cruciate ligament (ACL) and (b) distal epiphysis of the femur (eBone). Box and Whisker Plots represent the asymptomatic data per compartment (from left to right: purple, fast decay/short T_2_*; blue, slow decay/long T_2_*; red, water–fat mixture‐related (chemical shift); green, fat‐like). The medians of normalized weights computed for patients with a lesion in the considered region of interest (ROI) are presented with filled diamonds, while empty diamonds represent the median weights of patients with injuries only outside of the considered ROI. The same visualization is used in the top right graphs showing the ratios of long T_2_* to short T_2_* compartment weights. For the ACL, patients generally showed an increase in weight ratios relative to the asymptomatic volunteers, while for the eBone, weight shifts were ambiguous.

Median normalized weights for ACL are shown for the six asymptomatic volunteers, four patients with ACL lesions, and four patients without ACL lesions in Figure 7a. The median weights of the short and long T_2_* compartments for patients with and without ACL lesions were found outside of the asymptomatic IQRs (except for the long T_2_* compartment of one patient without ACL lesion). In general, patients showed a decreased contribution of the fast‐decaying signal and an increased contribution of the slowly‐decaying signal, except for one patient without ACL lesion who showed opposite weight shifts. The corresponding weight ratios enhanced this trend and displayed a clear distinction between ACLs of asymptomatic volunteers and patients. Regarding the fat‐like compartment, ACL patient weights either lay within the asymptomatic IQR or were higher than the asymptomatic outlier. In addition, the ACL patient weights of the water–fat mixture‐related compartment were generally lower than the asymptomatic median (Figure 7a).

Finally, the median weights for eBone were inspected (Figure 7b). Patients with acute eBone contusions (*n* = 2) showed increased short T_2_* and decreased long T_2_* compartment weights relative to the asymptomatic IQRs. Among the two patients with chronic knee pain and subchondral fractures in eBone, one patient had a highly increased weight ratio, while for the other patient, a weight ratio close to the asymptomatic median value was observed. For all subjects, median weights corresponding to the fat‐like and water–fat mixture‐related compartments remained below 0.10 and showed no distinctive changes for patients. Patients with no injuries in eBone (empty diamonds in Figure 7b) did not show a clear tendency relative to the asymptomatic data.

## 4 Discussion

In this study, we investigated the use of NMF for multi‐compartmental tissue characterization of knee structures from asymptomatic volunteers and patients with clinical indications for knee MRI. Overall, the convexity‐constrained NMF of 3D multi‐echo UTE MRI at 3‐T was found to be a promising tissue characterization technique due to its potential for tissue differentiation and its reproducibility. To the best of our knowledge, this is the first study to employ the unsupervised factorization method NMF in the context of knee qMRI and, more specifically, to characterize knee structures in a data‐driven manner based on 3D multi‐echo UTE MRI.

Prior to the NMF, the noise characteristics of the input data were considered given the use of a Frobenius norm for NMF, which assumes Gaussian distributed noise. The acquired complex‐valued UTE data underwent complex‐domain PCA denoising, which substantially reduces the Rician noise floor of the corresponding magnitude images by suppressing complex Gaussian noise before magnitude reconstruction [40]. As a result, the complex‐denoised magnitude data was much closer to being Gaussian distributed than Rician distributed, which justified the convexity‐constrained NMF in its applied form.

The factorization rank k was empirically determined to be 4, which corresponds to the value previously reported for the factorization of multi‐echo GRE brain data [27]. Basis functions and weight maps for factorization ranks k=3 and k=5 are additionally included in the Supporting Information (Figures S11 and S12). For the chosen rank, the discarded residual signal is small as shown in the residual maps computed for an asymptomatic volunteer and a patient volunteer (Figures S8 and S9, respectively). A more robust choice of the rank is known to be challenging as no established determination procedure exists [23]. Methods such as cross‐validation and statistical approaches including the Akaike's Information Criterion and the Bayesian Information Criterion have previously been considered, but they have shown a lack of agreement and their validity in the context of NMF has been questioned [25, 41].

The basis functions obtained with the NMF are comprised of two predominantly decaying signal curves associated with exponential T_2_* relaxation, even though no constraints related to such relaxation characteristics were imposed, in contrast to previous NMF studies which employed monotonicity constraints or Hankelization of the input data [22, 27]. To relate these signal features to the more familiar T_2_* mapping analysis, mono‐exponential curves were fitted to the raw basis function values of the predominantly decaying components, yielding time constants of 3.66 and 21.41 ms—values in line with characteristic short and long T_2_* relaxation behaviors of knee tissues [8]. It is worth highlighting that the computed time constants for these basis functions cannot be considered a direct quantification of the tissues' true T_2_* relaxation properties, as is the case for conventional model‐based analysis. To better understand the similarities between NMF and bi‐exponential signal modelling, bi‐exponential T_2_* maps were generated from the same UTE datasets and analyzed in a similar manner as the NMF results (Figures S2, S4, S5a, S5b, S5c, S6a, S6b, S7a, S7b, and S10). This supplementary analysis indicates that the NMF‐based decomposition, especially components 1 and 2, exhibits similar capabilities to bi‐exponential mapping in characterizing knee tissues and distinguishing between asymptomatic and lesioned knee structures (Figures S2, S3a, S3b, S5a, S5b, S5c, S6a, and S6b). However, we noted that the visual aspect of the NMF weight maps seems more anatomical than the noisier bi‐exponential fraction maps. The NMF weight maps thus appear more directly interpretable, with potentially higher clinical value given that NMF also provides information on more than two components. Furthermore, on a workstation equipped with an Intel Xeon w7‐2495X CPU (4.8 GHz), and an NVIDIA RTX A4000 GPU, the full convex NMF of a single dataset required 33 s. When assuming precomputed basis functions, this reduced to just 1 s. In comparison, the bi‐exponential model fitting on the same machine and dataset required 111 s. NMF thus exhibits computation times that are faster than bi‐exponential analysis and support its potential for clinical applicability.

The oscillatory basis function of component 3 was linked to signal from a water–fat mixture given its high correspondence to a simulated water–fat mixed signal sampled at the study's TEs (Figures S1a and S1b) as well as the high signal contribution of this component in regions typically affected by the chemical shift effect [42]. The apparent variation in oscillation frequency of this basis function arises from the non‐uniform sampling of in‐phase, out‐of‐phase, and intermediate TEs. Furthermore, it is important to note that as this component stems from archetypal voxels associated with a very specific water–fat mixture, it cannot be used to generally characterize partial volume content.

The basis function of component 4 was related to an amplitude‐modulated fat signal at 3‐T [43]. However, the expected decay due to natural dephasing of the latter signal could not be observed and even a slight upward trend over the considered TE range was noted, suggesting the presence of a residual signal of artifactual nature. It is important to note that fat suppression was not applied in this study as the effects of fat saturation pulses on the overall T_2_* relaxation behavior remain ambiguous [15, 42, 44]. Moreover, to more completely characterize knee structures, the suppression of any tissue type contributing to the overall signal (e.g., adipose tissue) was avoided.

Overall, the relative contributions of signal components in asymptomatic volunteers showed low inter‐subject variability, and the weights of the fast‐ and the slowly‐decaying signals were found to correspond with findings from in vivo UTE T_2_*‐based relaxometry studies. More specifically, PCL, PT, menisci, and bone displayed predominantly fast‐decaying behavior, consistent with the short T_2_* relaxation times (i.e., 1–10 ms) or predominant short T_2_* components (in the case of bi‐exponential modeling) reported in the literature for these structures [9, 11, 17, 45, 46]. PC and muscle showed a dominant contribution of the slowly decaying basis function in agreement with the long T_2_* relaxation behavior (i.e., T_2_* > 10 ms) previously documented for these tissues [8, 47]. The ACL exhibited a predominant long T_2_* component together with an important contribution of the short T_2_* component, as also previously observed [17]. The signal in HFP was mainly attributed to the fat‐like and short T_2_* components, which can be directly related to HFP composition (i.e., a lobular organization of white adipose tissue with collagenic stroma) [48]. While the presence of the fat‐like component in muscle and bone ROIs can be biologically motivated, its contribution to the signal in ACL and PC is rather anomalous. Yet, the presence of fat in the intercruciate space and the extension of the HFP around the patellar margins together with potential segmentation inaccuracies may explain the minor and elevated contributions of the fat‐like component in asymptomatic ACL and PC ROIs, respectively. Finally, the water–fat mixture‐related component had a marked presence in ACL, PCL, aLM, and PC. Chemical shift information has previously been quantified with UTE MRI in Achilles tendons, menisci, and cortical bone and has mainly been attributed to lipids [19, 20, 49]. However, given the histological absence of a substantial fat component in healthy tendons, ligaments, menisci, and cartilage, the presence of specific proteoglycans (such as leucin‐riche proteoglycans) and susceptibility differences between fiber groups in menisci can also be related to this off‐resonance signal compartment [20, 49]. Future work is needed to fully unravel the origins of the water–fat mixture‐related component.

The relatively higher variability across medians observed for the long T_2_* compartments of the PCL, aLM, and pLM in asymptomatic volunteers may, in part, be related to the magic angle effect (MAE) [50], as this effect has been shown to mainly affect the long T_2_* component in bi‐exponential modeling studies [8, 12, 51], and because fiber orientation was not explicitly controlled or approximated during ROI placement. The use of more insensitive acquisition techniques such as UTE adiabatic T1 ρ and magnetization transfer MRI could reduce the impact of the MAE [9, 52, 53].

Overall, lesioned meniscal and ligamentous structures displayed lower weights for the short T_2_* component and higher weights for the long T_2_* component compared to the asymptomatic datasets. Previous studies have documented similar increases of the T_2_* value for degenerated and torn menisci, and reconstructed ACLs [11, 45, 54, 55]. The deviations from the asymptomatic datasets were pronounced for injured pMMs and ACLs, and similar but weaker trends were recorded for injured aLMs and pLMs. The observed difference between medial and lateral meniscus horns may be related to well‐known age‐ and biomechanics‐related patterns of meniscal injuries [56]. In addition, weight ratios outside of the asymptomatic range for the pMM, pLM, and/or ACL ROIs were observed for patients with lesions in other knee structures. These observations may be concomitant with a more general inflammatory response in the knee joint upon localized structural damage and may even be indicative of a widespread sub‐clinical degeneration (i.e., beyond the direct location of the tear) [57, 58]. In contrast to the trends observed for menisci and ACL, differences in weights between deranged and asymptomatic eBone were found to be ambiguous, likely due to differences between acute and chronic injuries.

Despite the novelty and potential of the proposed method, the study has several limitations. First, although convexity‐constrained NMF promotes the extraction of archetypal voxel signals, the resulting components do not necessarily correspond to pure proton pools or distinct knee tissue compartments, but rather represent mixtures of such signal contributions, which may hinder their interpretability. Nevertheless, the presented parts‐based decomposition could be associated with biophysical signal trends and enabled differentiation between asymptomatic and impaired knee structures, particularly through components 1 and 2. Components 3 and 4 showed more ambiguous trends and consequently, their clinical relevance requires further investigation. Second, the number of included patients with pathological findings was limited and the analysis was only conducted for specific ROIs (menisci, ACL, and eBone) but, given the exploratory nature of the study, this was deemed sufficient to illustrate the potential of the proposed method. Moreover, the study included a limited amount of TEs per dataset due to hard‐coded restrictions of the UTE sequence and the requirement of clinically acceptable scan times. Yet, the acquisition of six TEs in this study appeared to be sufficient for an adequate tissue characterization given the demonstrated reproducibility of the basis functions across asymptomatic subjects, the overall low variability of median compartment weights and the discriminating potential between asymptomatic and symptomatic datasets for selected ROIs. Finally, findings from the NMF analysis were contrasted with published in vivo UTE T_2_*‐based relaxometry results, and a supplementary bi‐exponential T_2_* mapping analysis of the acquired 3D multi‐echo UTE MRI data was performed for direct comparison of the data‐driven convexity‐constrained NMF with a conventional model‐based technique. While signal models with more than three compartments have previously been used in musculoskeletal UTE MRI studies [9, 20], it remains difficult to determine an accurate signal model parametrization for the data at hand, also considering the partial volume effect and the presence of noise. In addition, advanced parameter estimation techniques considering prior knowledge would be required to manage the resulting ill‐conditioned optimization problem [21].

As NMF is a data‐driven technique, a direction for future work is to evaluate the sensitivity of the decomposition to variations in acquisition parameters and sequence design. While in this study, the resulting basis functions and weight maps showed consistency across subjects and across datasets with two different TRs and sets of TEs, differences in sequence design, acquisition parameters (e.g., TE sampling scheme) and scanner hardware may influence which components can be extracted reliably. Therefore, systematic investigations across acquisition protocols, scanners and sites will be important to assess the robustness of NMF of knee MRI. Although NMF was only applied to magnitude images in this study, as convexity‐constrained NMF is not directly compatible with complex‐valued data, the exploration of complex NMF approaches may also constitute an interesting direction for future work [59].

Another suggestion for future work is the application of NMF to UTE data of isolated knee structures from both asymptomatic and deranged knees to obtain more tissue‐specific decompositions with potentially more representative basis functions reflecting the biochemical composition and microstructure of a particular tissue and/or lesion type. This may also facilitate differentiation based on clinical indication and/or grades of structural defects. To support these potential clinical applications, the proposed NMF‐based approach should also be assessed in a large patient cohort with arthroscopic correlation.

In conclusion, convexity‐constrained NMF of 3D multi‐echo UTE MRI of the knee at 3‐T can extract biophysically related signal components and describe their relative contribution to the measured MRI signal in various knee tissues. Moreover, the proposed framework allows to differentiate between asymptomatic and impaired knee structures. Thus, the presented data‐driven method acts as a promising technique for quantitative characterization of knee structures and identification of quantitative imaging biomarkers of tissue disease and degeneration, and consequently shows potential for improved diagnosis and monitoring of internal knee derangements.

## Author Contributions

Conceptualization and design (Céline Smekens, Thomas Janssens, Ben Jeurissen), data acquisition (Céline Smekens), software (Céline Smekens, Ben Jeurissen, Patrick S. Fuchs), data analysis (Céline Smekens, Pieter Van Dyck, Ben Jeurissen, Patrick S. Fuchs), data interpretation (Céline Smekens, Pieter Van Dyck, Jan Sijbers, Thomas Janssens, Ben Jeurissen, Patrick S. Fuchs), drafting of the manuscript (Céline Smekens), review and editing of the manuscript (Céline Smekens, Pieter Van Dyck, Jan Sijbers, Thomas Janssens, Ben Jeurissen, Patrick S. Fuchs), supervision (Thomas Janssens, Ben Jeurissen). All authors read and approved the submitted manuscript.

Thomas Janssens and Ben Jeurissen jointly supervised this work and therefore share last authorship.

## Funding

This work was supported by Horizon 2020, MSCA, 764513; Universiteit Antwerpen, 44883; Fonds Wetenschappelijk Onderzoek, G058723N and G096324N; Horizon Europe, European Research Council, ADAMI ‐ 101126235. Views and opinions expressed are those of the authors only and do not necessarily reflect those of the European Union or European Research Council. Neither the European Union nor the granting authority can be held responsible for them.

## Conflicts of Interest

Two authors (Céline Smekens and Thomas Janssens) are employees of Siemens Healthcare NV/SA, Belgium. This employer had no role in the design, implementation, or analysis of this research study. The other authors declare no conflicts of interest.

## Supporting information


**Figure S1:** Simulated continuous (blue) MR magnitude signals of a voxel with water–fat mixture (proton density: 50% water–50% fat) and simulated discrete (red) MR magnitude signal, sampled at echo times (TEs) used in the (a) pilot study and (b) main study.
**Figure S2:** Sagittal 3D T2 FS CAIPIRINHA SPACE TSE images and 𝒇_𝑺_ and 𝒇_𝑳_ maps, respectively corresponding to the fraction of the short T_2_* component and the fraction of the long T_2_* component, of the left knee of a 23‐year‐old female asymptomatic volunteer (top row), the right knee of a 33‐year‐old female patient with sustained knee pain (middle row) and the left knee of a 72‐year‐old female patient reporting episodes of knee giving away (bottom row). No lesions were observed in the posterior horn of the medial meniscus (pMM) of the 33‐year‐old female patient on anatomical MR images, while a complex tear was noted in the pMM of the 72‐year‐old female patient (white arrows). The lesioned pMM showed lower fractions (darkening) in the 𝒇_𝑺_ map and increased fractions (brightening) in the 𝒇_𝑳_ map in comparison to the maps of the asymptomatic volunteer. For the female patient with no detected pMM lesion, a slight darkening in the 𝒇_𝑺_ map and shallow brightening in the 𝒇_𝑳_ map can be observed for the pMM region.
**Figure S3:** Scatter plots displaying the correlation between (a) NMF weights for component 1 (i.e., fast‐decaying/short T_2_*‐related component) and the fractions of the short T_2_* component 𝒇_𝑺_, and (b) NMF weights for component 2 (i.e., slowly‐decaying/long T_2_*‐related component) and the fractions of long T_2_* component 𝒇_𝑳_, for a region‐of‐interest in the posterior medial meniscus (pMM) of a 23‐year‐old female asymptomatic volunteer (Asymptomatic, red), a 33‐year‐old female patient with sustained knee pain but no observed lesions in the pMM (Patient 2, green), and a 72‐year‐old female patient with a complex tear in the pMM (Patient 1, blue). Spearman correlation coefficients of 0.84 and of 0.87 were obtained for the data in (a) and (b), respectively.
**Figure S4:** Sagittal 3D T2 FS CAIPIRINHA SPACE TSE images and 𝑻𝟐_𝑺_* and 𝑻𝟐_𝑳_* maps, respectively corresponding to the short T_2_* relaxation time constant and the long T_2_* relaxation time constant, of the left knee of a 23‐year‐old female asymptomatic volunteer (top row), the right knee of a 33‐year‐old female patient with sustained knee pain (middle row) and the left knee of a 72‐year‐old female patient reporting episodes of knee giving away (bottom row). No lesions were observed in the posterior horn of the medial meniscus (pMM) of the 33‐year‐old female patient on anatomical MR images, while a complex tear was noted in the pMM of the 72‐year‐old female patient (white arrows). Compared to the maps of the asymptomatic volunteer, the lesioned pMM (bottom row) does not show a clear difference in the 𝑻𝟐_𝑺_* map, while the 𝑻𝟐_𝑳_* map does display increased 𝑻𝟐_𝑳_* values in the pMM.
**Figure S5:** Median fractions of the short T_2_* component 𝒇_𝑺_ and of the long T_2_* component 𝒇_𝑳_ for the menisci of asymptomatic volunteers and patients. Three meniscal regions are considered: (a) posterior horn of medial meniscus (pMM), (b) anterior horn of lateral meniscus (aLM), and (c) posterior horn of lateral meniscus (pLM). Box and Whisker Plots represent the distribution of the median fractions of the 6 asymptomatic volunteers for 𝒇_𝑺_ and 𝒇_𝑳_. The medians of the fractions for patients with meniscus lesions are presented individually (filled diamonds) next to the medians for patients with injuries solely outside of the considered meniscus region of interest (empty diamonds). Similar data visualization is used in the top right graphs which represent ratios of median 𝒇_𝑳_ to median 𝒇_𝑺_ values. Overall, lesioned menisci display a trend toward lower 𝒇_𝑺_ values and higher 𝒇_𝑳_ values, which is highlighted by increased ratios for the patients (especially for the pMM and pLM ROIs).
**Figure S6:** Median fractions of the short T_2_* component 𝒇_𝑺_ and of the long T_2_* component 𝒇_𝑳_ of asymptomatic volunteers and patients for the (a) anterior cruciate ligament (ACL) and (b) distal epiphysis of the femur (eBone). Box and Whisker Plots represent the distribution of the median fractions of the 6 asymptomatic volunteers for 𝒇_𝑺_ and 𝒇_𝑳_. The medians of the fractions for patients with a lesion in the considered region of interest (ROI) are presented with filled diamonds, while empty diamonds represent the median fractions of patients with injuries only outside of the considered ROI. The same data visualization is used in the top right graphs which represent ratios of median 𝒇_𝑳_ to median 𝒇_𝑺_ values. For the ACL, patients generally showed an increase in the 𝒇_𝑳_ to 𝒇_𝑺_ ratios relative to the asymptomatic volunteers, while for the eBone, weight shifts were ambiguous.
**Figure S7:** Medians of the short (a) and long (b) T_2_* relation time constants (i.e., 𝑻𝟐_𝑺_* and 𝑻𝟐_𝑳_*, respectively) of asymptomatic volunteers and patients for the anterior horn of lateral meniscus (aLM), posterior horn of lateral meniscus (pLM), posterior horn of medial meniscus (pMM), anterior cruciate ligament (ACL) and distal epiphysis of the femur (eBone). Box and Whisker Plots represent the distribution of the median relaxation time constants of the 6 asymptomatic volunteers. The medians of the relaxation time constants for patients with a lesion in the considered region of interest (ROI) are presented with filled diamonds, while empty diamonds represent the median relaxation time constants of patients with injuries only outside of the considered ROI.
**Figure S8:** Residual analysis of the convexity‐constrained NMF applied to the UTE dataset of a 23‐year‐old female asymptomatic volunteer (left knee). Component‐specific signal intensity maps reconstructed from the NMF factors are shown next to the corresponding residual map (columns) for each echo time (TE) (rows). The upper limit of the corresponding unitless signal intensity (SI) scale was set to the rounded median of the maximum signal intensity values of the component intensities at the given TE.
**Figure S9:** Residual analysis of the convexity‐constrained NMF applied to the UTE dataset of a 46‐year‐old female patient volunteer (left knee, pivot‐shift injury). Component‐specific signal intensity maps reconstructed from the NMF factors are shown next to the corresponding residual map (columns) for each echo time (TE) (rows). The upper limit of the corresponding unitless signal intensity (SI) scale was set to the rounded median of the maximum signal intensity values of the component intensities at the given TE.
**Figure S10:** Residual analysis of the convexity‐constrained NMF and bi‐exponential T_2_* mapping applied to the UTE dataset of a 23‐year‐old female asymptomatic volunteer (left side) and a 46‐year‐old female patient volunteer with pivot‐shift injury (right side). Residual maps of the NMF are shown next to the corresponding residual maps of the biexponential model fit (columns) for each echo time (TE) (rows). The upper limit of the corresponding unitless signal intensity (SI) scale was set to the rounded median of the maximum signal intensity values of the component intensities at the given TE.
**Figure S11:** Basis functions resulting from the convexity‐constrained NMF applied to the large dataset from the pilot study for a rank 𝑘 of 3, 4 and 5. The shown basis functions are normalized to the maximal signal intensity (at TE_1_).
**Figure S12:** Weight maps resulting from the convexity‐constrained NMF applied to the large dataset from the pilot study for a rank 𝑘 of 3, 4 and 5. The weight maps are normalized to sum up to 1.

## Data Availability

The data that support the findings of this study are available from the corresponding author upon reasonable request.
