# Supplementary material for: Data‐Driven Characterization of Knee Structures Using Non‐Negative Matrix Factorization of 3D Multi‐Echo UTE MRI
Source: NMR Biomed. 2026 May 7;39:e70299. doi: 10.1002/nbm.70299 (PMC13150805; doi:10.1002/nbm.70299)
Supplement: Supplementary file 1 — Figure S1: Simulated continuous (blue) MR magnitude signals of a voxel with water–fat mixture (proton density: 50% water–50% fat) and simulated discrete (red) MR magnitude signal, sampled at echo times (TEs) used in the (a) pilot study and (b) main study. Figure S2: Sagittal 3D T2 FS CAIPIRINHA SPACE TSE images and 𝒇𝑺 and 𝒇𝑳 maps, respectively corresponding to the fraction of the short T2* component and the fraction of the long T2* component, of the left knee of a 23‐year‐old female asymptomatic volunteer (top row), the right knee of a 33‐year‐old female patient with sustained knee pain (middle row) and the left knee of a 72‐year‐old female patient reporting episodes of knee giving away (bottom row). No lesions were observed in the posterior horn of the medial meniscus (pMM) of the 33‐year‐old female patient on anatomical MR images, while a complex tear was noted in the pMM of the 72‐year‐old female patient (white arrows). The lesioned pMM showed lower fractions (darkening) in the 𝒇𝑺 map and increased fractions (brightening) in the 𝒇𝑳 map in comparison to the maps of the asymptomatic volunteer. For the female patient with no detected pMM lesion, a slight darkening in the 𝒇𝑺 map and shallow brightening in the 𝒇𝑳 map can be observed for the pMM region. Figure S3: Scatter plots displaying the correlation between (a) NMF weights for component 1 (i.e., fast‐decaying/short T2*‐related component) and the fractions of the short T2* component 𝒇𝑺, and (b) NMF weights for component 2 (i.e., slowly‐decaying/long T2*‐related component) and the fractions of long T2* component 𝒇𝑳, for a region‐of‐interest in the posterior medial meniscus (pMM) of a 23‐year‐old female asymptomatic volunteer (Asymptomatic, red), a 33‐year‐old female patient with sustained knee pain but no observed lesions in the pMM (Patient 2, green), and a 72‐year‐old female patient with a complex tear in the pMM (Patient 1, blue). Spearman correlation coefficients of 0.84 and of 0.87 were obtained for the data in [file NBM-39-e70299-s001.pdf]

Supplementary Material

S1. Simulation of water-fat mixed signal (chemical shift effect)

To investigate the interpretability of the basis function of component 3, a complex multi-echo UTE signal considering combined contributions of water and fat proton pools was simulated. The total signal was defined as the sum of a mono-exponentially decaying water component and multiple off-resonance fat components. Water was referenced at 4.67 ppm [1], while fat was modeled using a standard 6-peak spectrum [2]. Chemical shift frequency offsets were computed from the water-fat ppm differences and the Larmor frequency at the scanner's field strength. Distinct representative T_2* values were assigned to water and fat (i.e., 25 ms and 10 ms, respectively [3]), and equal proton densities were assumed. A densely sampled echo time (TE) range (0-26 ms) was used to generate a continuous signal driven by T_2* relaxation and chemical shift, while two discrete sets of TEs corresponding to the sets used in the pilot and main study protocols were used to simulate the signal at the sampled TEs. The magnitude component of the simulated signals is displayed in **Figure S1**.

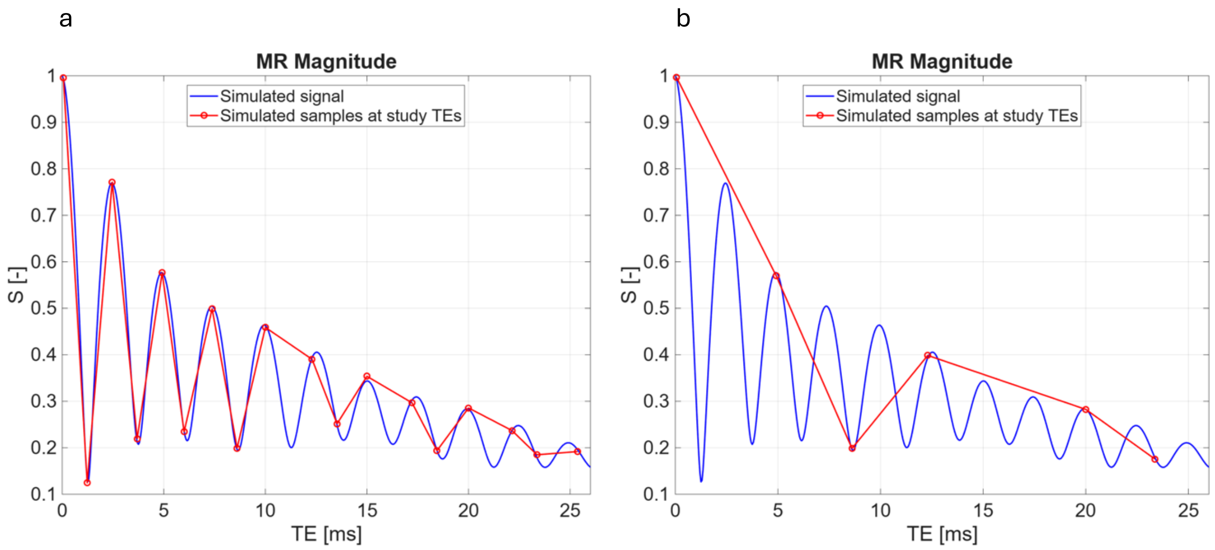

Figure S1. *Simulated continuous (blue) MR magnitude signals of a voxel with water-fat mixture (proton density: 50% water-50% fat) and simulated discrete (red) MR magnitude signal, sampled at echo times (TEs) used in the (a) pilot study and (b) main study.*

When comparing the simulated magnitude signals sampled at the selected TEs (red curves in **Figure S1a** and **Figure S1b**) to the NMF-estimated basis functions of component 3, a high correspondence can be observed for both the pilot study (**Figure 3a**) and the main study (**Figure 3b**). This justifies the attribution of component 3 to an archetype representative of water-fat mixed signal.

S2. Bi-exponential T_2* mapping analysis

A bi-exponential T_2* mapping analysis was performed using the pre-processed UTE T_2*-weighted datasets for comparison with the NMF weight maps. The model equation used for the voxel-wise bi-exponential T_2* mapping was:

$$\mathbf{S(t)} = \mathbf{A}_{s}\boldsymbol{e}^{\left(-\frac{\mathbf{t}}{\boldsymbol{T2}_{S}^{*}}\right)} + \mathbf{A}_{L}\boldsymbol{e}^{\left(-\frac{\mathbf{t}}{\boldsymbol{T2}_{L}^{*}}\right)}$$

where $\mathbf{S}(\mathbf{t})$ is the time-dependent signal intensity, $\boldsymbol{T2}_{S}^{*}$ and $\boldsymbol{T2}_{L}^{*}$ are the T_2* relaxation time constants, respectively associated with the fast-relaxing macromolecular bound water component and the slowly-relaxing bulk water component, and $\mathbf{A}_{S}$ and $\mathbf{A}_{L}$ are the signal amplitude of the short and long T_2* components, respectively. Noise was not modeled as the datasets previously underwent complex denoising. The fitting boundaries for $\boldsymbol{T2}_{S}^{*}$ and $\boldsymbol{T2}_{L}^{*}$ were defined as [0 ms, 10 ms] and [10 ms, 1000 ms], respectively. The fraction of the short T_2* component $\mathbf{f}_{S}$ was defined as $\boldsymbol{A}_{S}/(\boldsymbol{A}_{S} + \boldsymbol{A}_{L})$, while the fraction of the long T_2* component $\mathbf{f}_{L}$ was defined as $\boldsymbol{A}_{L}/(\boldsymbol{A}_{S} + \boldsymbol{A}_{L})$. Parameter maps were computed for $\mathbf{f}_{S}$, $\mathbf{f}_{L}$, $\boldsymbol{T2}_{S}^{*}$ and $\boldsymbol{T2}_{L}^{*}$.

The fitting of the bi-exponential model to the pre-processed UTE T_2*-weighted datasets was performed using a conventional voxel-wise nonlinear-least-square optimization with the askAdam solver incorporated in the MATLAB package GACELLE [4].

While NMF weight maps (matrix factor $\boldsymbol{H}$) provide information on voxel volume fractions, $\mathbf{f}_{S}$ and $\mathbf{f}_{L}$ maps represent signal fraction maps. To compare the information of the NMF weight maps (components 1 and 2) and the results from the bi-exponential T_2* mapping, $\mathbf{f}_{S}$ and $\mathbf{f}_{L}$ maps of an asymptomatic volunteer and two patients are presented in **Figure S2**. These fraction maps belong to the same asymptomatic volunteer and patients considered for the NMF weight maps presented in **Figure 5** to facilitate the comparison. Moreover, the NMF weights (components 1 and 2) and bi-exponential fractions extracted from a region-of-interest (ROI) in the posterior medial meniscus of the same 3 volunteers are contrasted by means of scatter plots in **Figure S3**. Finally, the $\boldsymbol{T2}_{S}^{*}$ and $\boldsymbol{T2}_{L}^{*}$ maps estimated for these volunteers are also presented in **Figure S4.**

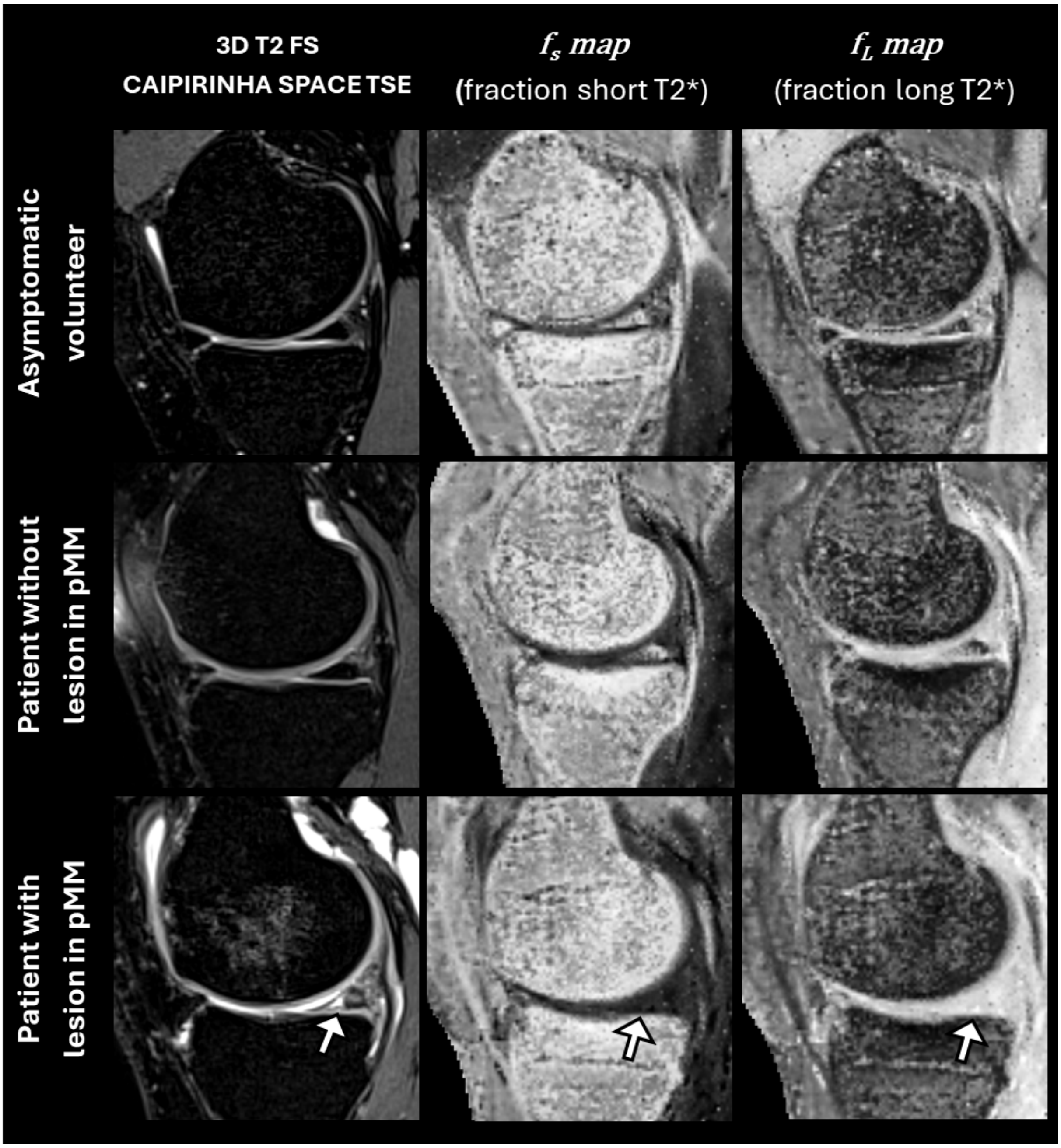

Figure S2. *Sagittal 3D T_2 FS CAIPIRINHA SPACE TSE images and f_S and f_L maps, respectively corresponding to the fraction of the short T_2* component and the fraction of the long T_2* component, of the left knee of a 23-year-old female asymptomatic volunteer (top row), the right knee of a 33-year-old female patient with sustained knee pain (middle row) and the left knee of a 72-year-old female patient reporting episodes of knee giving away (bottom row). No lesions were observed in the posterior horn of the medial meniscus (pMM) of the 33-year-old female patient on anatomical MR images, while a complex tear was noted in the pMM of the 72-year-old female patient (white arrows). The lesioned pMM showed lower fractions (darkening) in the f_S map and increased fractions (brightening) in the f_L map in comparison to the maps of the asymptomatic volunteer. For the female patient with no detected pMM lesion, a slight darkening in the f_S map and shallow brightening in the f_L map can be observed for the pMM region.*

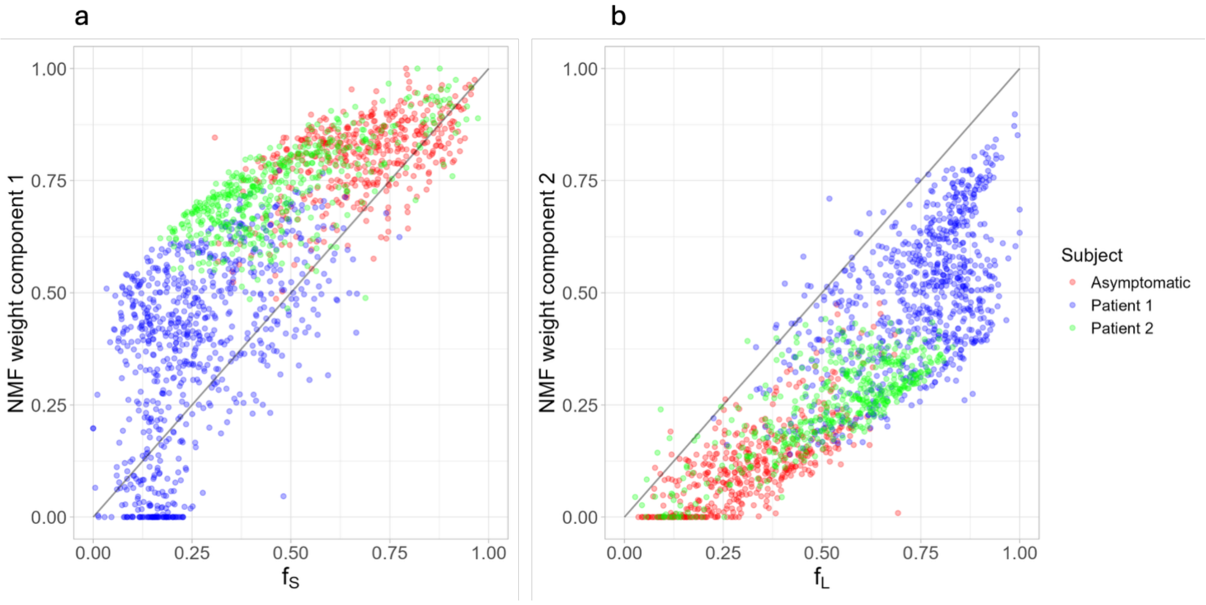

Figure S3. *Scatter plots displaying the correlation between (a) NMF weights for component 1 (i.e., fast-decaying/short T_2*-related component) and the fractions of the short T_2* component* $\boldsymbol{f_S}$*, and (b) NMF weights for component 2 (i.e., slowly-decaying/long T_2*-related component) and the fractions of long T_2* component* $\boldsymbol{f_L}$*, for a region-of-interest in the posterior medial meniscus (pMM) of a 23-year-old female asymptomatic volunteer (Asymptomatic, red), a 33-year-old female patient with sustained knee pain but no observed lesions in the pMM (Patient 2, green), and a 72-year-old female patient with a complex tear in the pMM (Patient 1, blue). Spearman correlation coefficients of 0.84 and of 0.87 were obtained for the data in (a) and (b), respectively.*

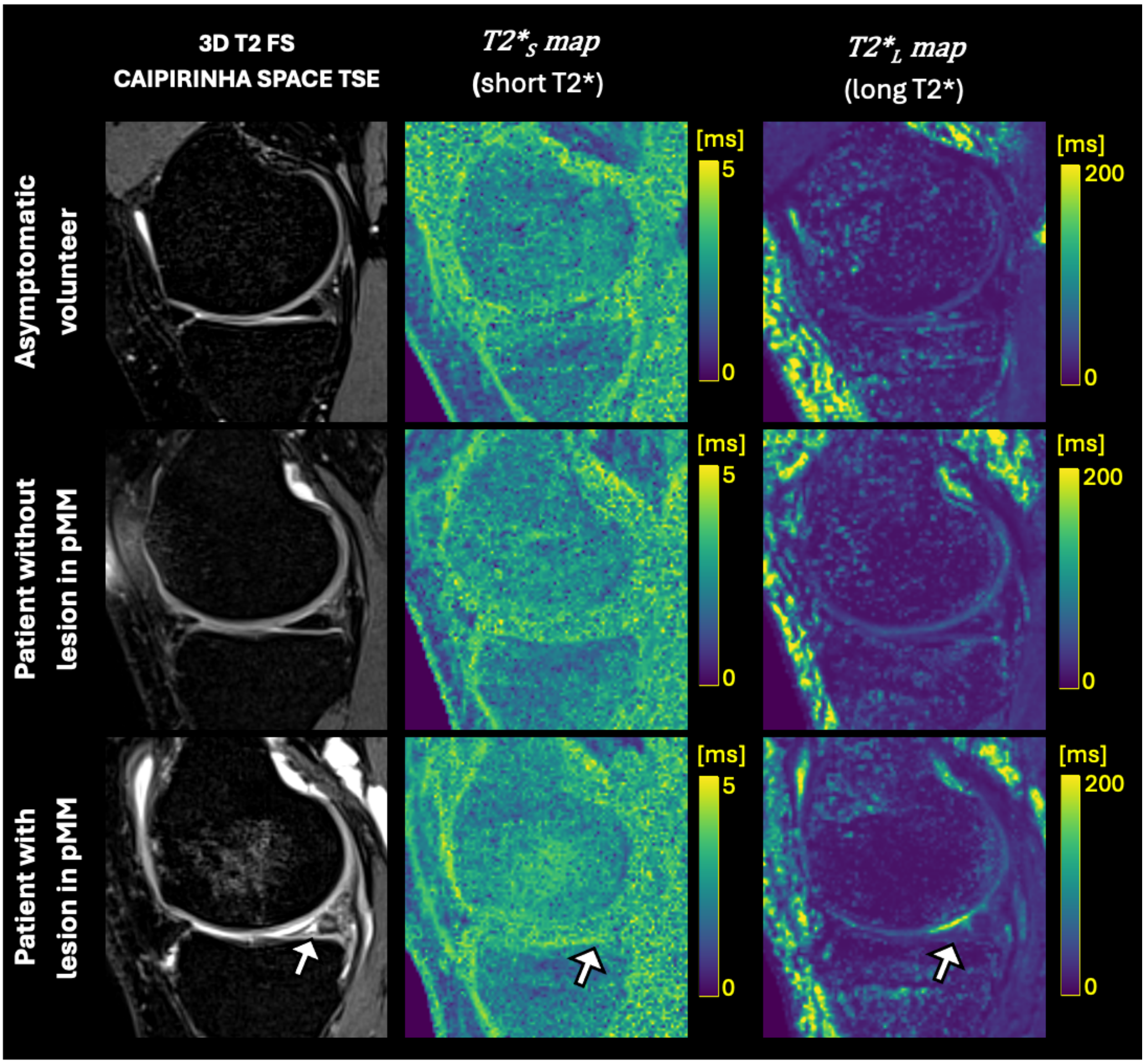

Figure S4. *Sagittal 3D T_2 FS CAIPIRINHA SPACE TSE images and $\mathbf{T2}_S^*$ and $\mathbf{T2}_L^*$ maps, respectively corresponding to the short T_2* relaxation time constant and the long T_2* relaxation time constant, of the left knee of a 23-year-old female asymptomatic volunteer (top row), the right knee of a 33-year-old female patient with sustained knee pain (middle row) and the left knee of a 72-year-old female patient reporting episodes of knee giving away (bottom row). No lesions were observed in the posterior horn of the medial meniscus (pMM) of the 33-year-old female patient on anatomical MR images, while a complex tear was noted in the pMM of the 72-year-old female patient (white arrows). Compared to the maps of the asymptomatic volunteer, the lesioned pMM (bottom row) does not show a clear difference in the $\mathbf{T2}_S^*$ map, while the $\mathbf{T2}_L^*$ map does display increased $\mathbf{T2}_L^*$ values in the pMM.*

Overall, the NMF weight maps of components 1 and 2 in **Figure 5** display similar trends to the $\mathbf{f_S}$ and $\mathbf{f_L}$ maps, respectively (**Figure S2**). Compared to maps of the asymptomatic reference, weight map $\mathbf{H_1}$ and fraction map $\mathbf{f_S}$ both show decreased values in the lesioned pMM, while weight map $\mathbf{H_2}$ and fraction map $\mathbf{f_L}$ display increased values in the affected region. Despite the observed similarities, the visual aspect of the NMF weight maps seem to correspond more to anatomical MR images than the noisier fraction maps. The NMF weight maps thus appear more directly interpretable, with potentially higher clinical value.

For the representative pMM structures shown in **Figure S2**, the correlation between the bi-exponential short T_2* fractions and the NMF weights of component 1 is high (Spearman correlation coefficient = 0.84, **Figure S3a**). A similarly high correlation (Spearman correlation coefficient = 0.87, **Figure S3b**) was observed between the bi-exponential long T_2* fractions and the NMF weights of component 2. Considerably higher correlations were not to be expected given the inherent differences in the nature of the fractions given by NMF (volume fractions) and the ones provided by the bi-exponential model (signal fractions). Interestingly, the scatter plots of the pMM (**Figure S3**) show clear shifts in values (weights/fractions) from asymptomatic to lesioned pMM with intermediate values for the symptomatic knee without pMM lesion.

In contrast to the fraction maps, the $\boldsymbol{T2_S^*}$ and $\boldsymbol{T2_L^*}$ maps (**Figure S4**) show less pronounced trends across asymptomatic and symptomatic knees. However, the $\boldsymbol{T2_L^*}$ map does highlight the lesioned pMM region with increased $T2_L^*$ values.

The ROIs previously defined in the menisci, anterior cruciate ligament (ACL) and the distal epiphysis of the femur (eBone) of asymptomatic volunteers and patients for the NMF analysis were also applied to the bi-exponential maps and analyzed in the same way as for NMF (see **Figure 6** and **Figure 7**). In **Figure S5** to **Figure S7** the differentiation potential between lesioned and asymptomatic knee structures using bi-exponential mapping is presented for the pMM, anterior horn of lateral meniscus (aLM), posterior horn of lateral meniscus (pLM), ACL and eBone. Median fraction values retrieved from the ROIs in the $\mathbf{f}_S$ and $\mathbf{f}_L$ maps are presented in **Figure S5** and **Figure S6**. Median T_2* values retrieved from the ROIs in the $\boldsymbol{T2_S^*}$ and $\boldsymbol{T2_L^*}$ maps are presented in **Figure S7**.

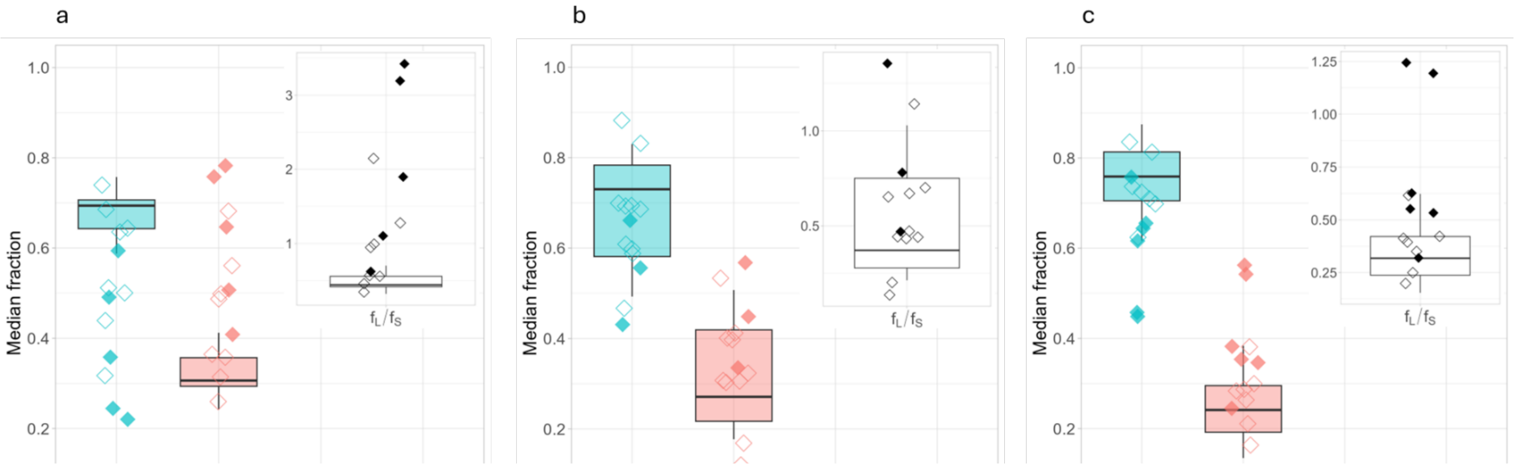

***Figure S5**. Median fractions of the short T_2* component $\boldsymbol{f_S}$ and of the long T_2* component $\boldsymbol{f_L}$ for the menisci of asymptomatic volunteers and patients. Three meniscal regions are considered: (a) posterior horn of medial meniscus (pMM), (b) anterior horn of lateral meniscus (aLM), and (c) posterior horn of lateral meniscus (pLM). Box and Whisker Plots represent the distribution of the median fractions of the 6 asymptomatic volunteers for $\boldsymbol{f_S}$ and $\boldsymbol{f_L}$. The medians of the fractions for patients with meniscus lesions are presented individually (filled diamonds) next to the medians for patients with injuries solely outside of the considered meniscus region of interest (empty diamonds). Similar data visualization is used in the top right graphs which represent ratios of median $\boldsymbol{f_L}$ to median $\boldsymbol{f_S}$ values. Overall, lesioned menisci display a trend toward lower $\boldsymbol{f_S}$ values and higher $\boldsymbol{f_L}$ values, which is highlighted by increased ratios for the patients (especially for the pMM and pLM ROIs).*

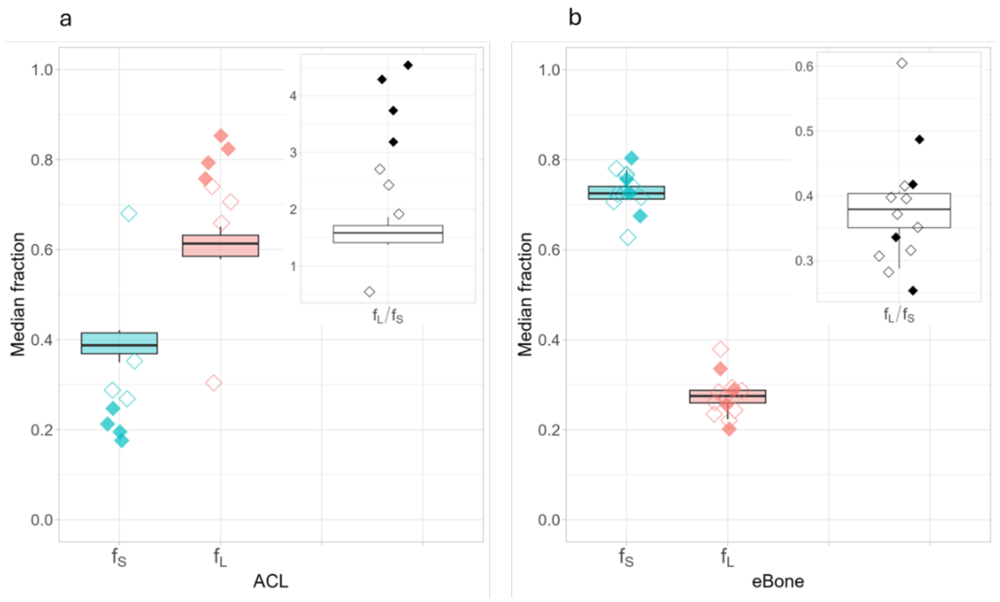

Figure S6. *Median fractions of the short T_2* component f_S and of the long T_2* component f_L of asymptomatic volunteers and patients for the (a) anterior cruciate ligament (ACL) and (b) distal epiphysis of the femur (eBone). Box and Whisker Plots represent the distribution of the median fractions of the 6 asymptomatic volunteers for f_S and f_L. The medians of the fractions for patients with a lesion in the considered region of interest (ROI) are presented with filled diamonds, while empty diamonds represent the median fractions of patients with injuries only outside of the considered ROI. The same data visualization is used in the top right graphs which represent ratios of median f_L to median f_S values. For the ACL, patients generally showed an increase in the f_L to f_S ratios relative to the asymptomatic volunteers, while for the eBone, weight shifts were ambiguous.*

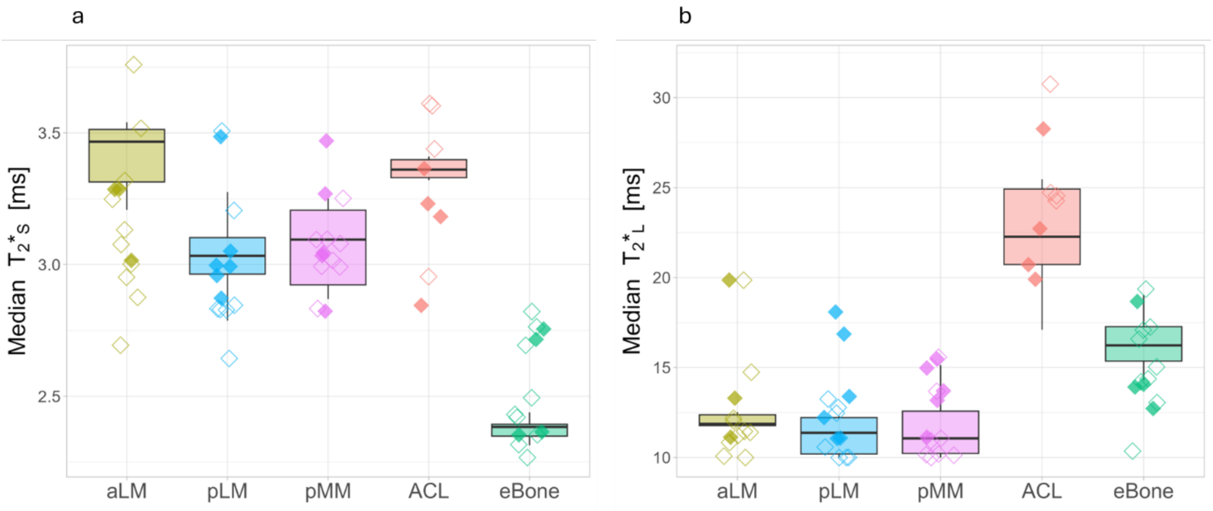

Figure S7. *Medians of the short (a) and long (b) T_2* relation time constants (i.e., $T2_S^*$ and $T2_L^*$, respectively) of asymptomatic volunteers and patients for the anterior horn of lateral meniscus (aLM), posterior horn of lateral meniscus (pLM), posterior horn of medial meniscus (pMM), anterior cruciate ligament (ACL) and distal epiphysis of the femur (eBone). Box and Whisker Plots represent the distribution of the median relaxation time constants of the 6 asymptomatic volunteers. The medians of the relaxation time constants for patients with a lesion in the considered region of interest (ROI) are presented with filled diamonds, while empty diamonds represent the median relaxation time constants of patients with injuries only outside of the considered ROI.*

The trends observed for the median short and long T_2* fractions ($\mathbf{f}_S$ and $\mathbf{f}_L$) across asymptomatic and patient volunteers are highly similar to the trends observed for the median NMF weights of components 1 (short T_2*) and 2 (long T_2*) for all considered ROIs

(see **Figure 6** and **Figure 7**). Compared to the median NMF weights of asymptomatic volunteers, the median fractions $\mathbf{f}_S$ and $\mathbf{f}_L$ of asymptomatic volunteers show a slightly higher variability for the menisci and a slightly lower variability for the ACL. While both NMF weights (components 1 and 2) and bi-exponential fractions exhibit a good capacity of differentiating lesioned tissues from asymptomatic ones, the median T_2* relation time constants show less clear trends between asymptomatic and lesioned tissues (**Figure S7**). The estimation of the $T2_S^*$ and $T2_L^*$ values is likely to have been affected by the use of in-phase, out-of-phase and intermediate TEs in this study given that no off-resonance components were considered in the model-based analysis.

S3. Residual analysis of the convexity-constrained NMF

The factorization rank k was empirically determined to be 4. To evaluate the corresponding factorization error, signal residuals $\boldsymbol{R}$ were computed based on the element-wise absolute difference between the acquired data matrix $\mathbf{V}$ and the reconstructed data matrix $\widehat{\mathbf{V}} = \mathbf{VWH}$, i.e.,

$$\mathbf{R} = \left|\mathbf{V} - \widehat{\mathbf{V}}\right| = |\mathbf{V} - \mathbf{VWH}|.$$

Figure S8 shows the resulting residual maps for an asymptomatic volunteer and **Figure S9** displays the results for a patient. Both figures show the residual maps of representative slices next to the corresponding component-specific signal intensity maps at each echo time (TE). To facilitate the visualization, the upper limit of the corresponding unitless intensity scale was set to the rounded median of the maximum signal intensity values of the component intensities at the given TE.

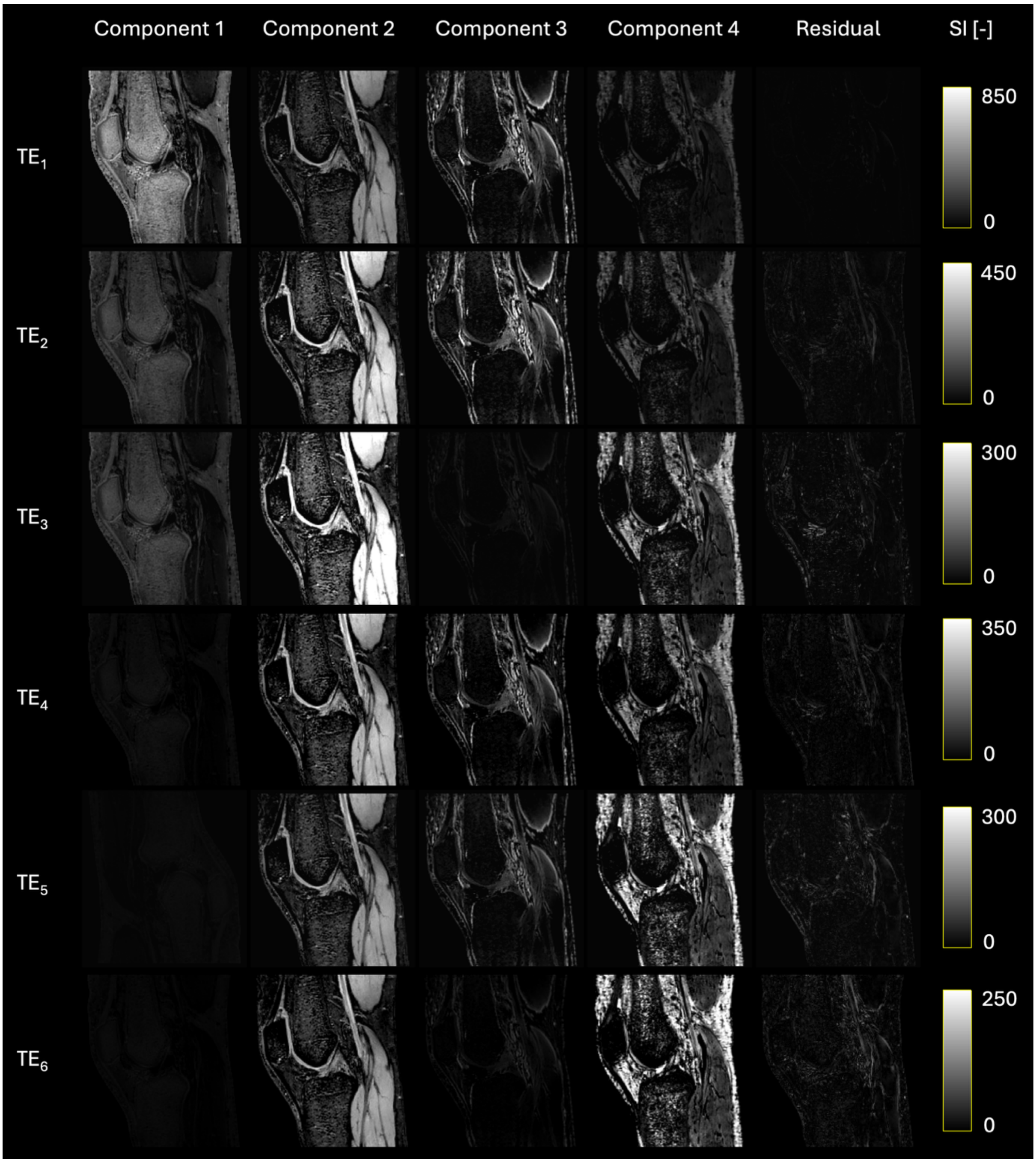

Figure S8. *Residual analysis of the convexity-constrained NMF applied to the UTE dataset of a 23-year-old female asymptomatic volunteer (left knee). Component-specific signal intensity maps reconstructed from the NMF factors are shown next to the corresponding residual map (columns) for each echo time (TE) (rows). The upper limit of the corresponding unitless signal intensity (SI) scale was set to the rounded median of the maximum signal intensity values of the component intensities at the given TE.*

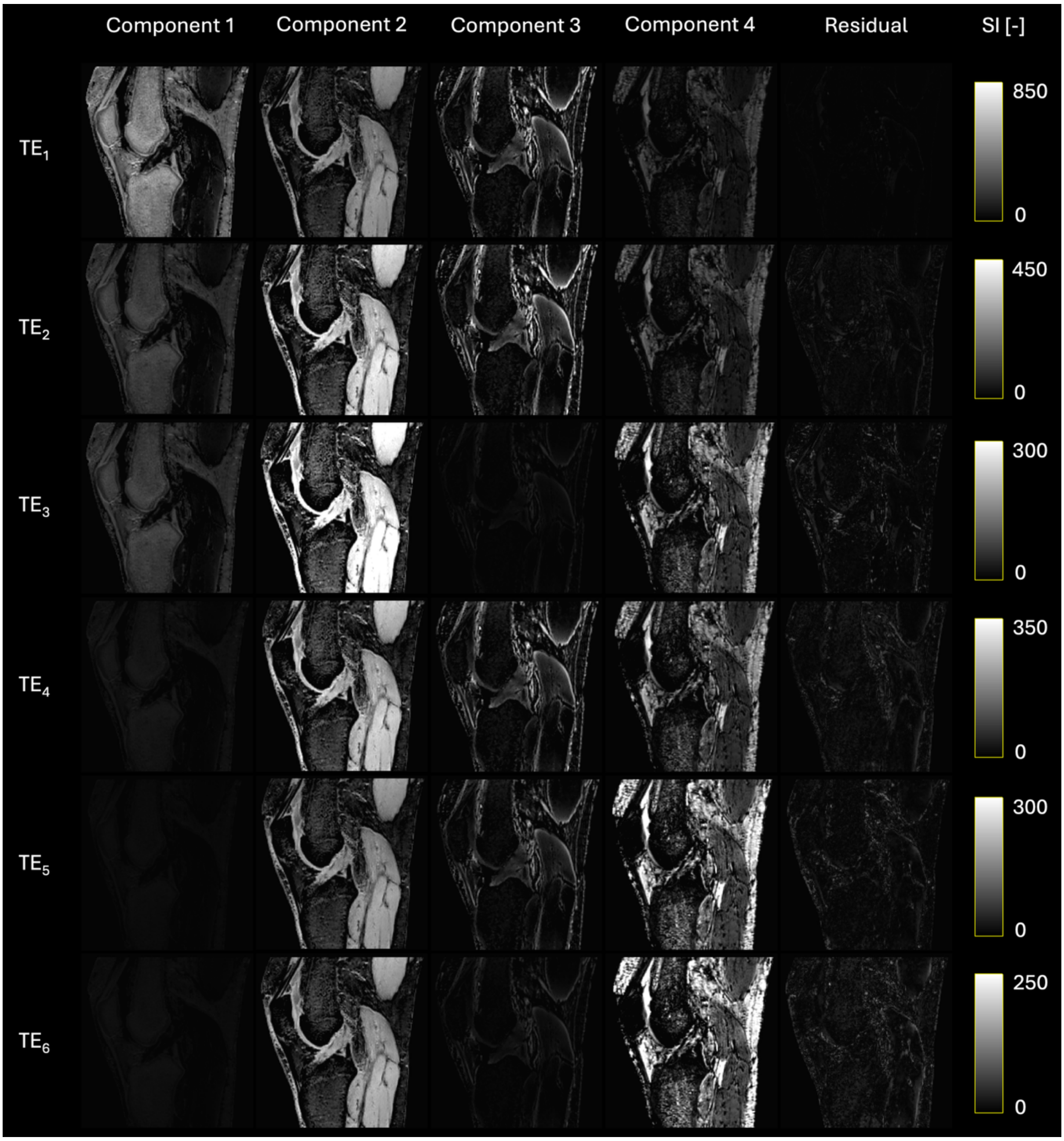

Figure S9. *Residual analysis of the convexity-constrained NMF applied to the UTE dataset of a 46-year-old female patient volunteer (left knee, pivot-shift injury). Component-specific signal intensity maps reconstructed from the NMF factors are shown next to the corresponding residual map (columns) for each echo time (TE) (rows). The upper limit of the corresponding unitless signal intensity (SI) scale was set to the rounded median of the maximum signal intensity values of the component intensities at the given TE.*

For both subjects, the residuals of the convexity-constrained NMF at TE_1 contain little to no visible signal, compared to the other components. More specifically, at TE_1, the median relative residuals (with respect to the total acquired signal) are 0.2% and 0.3% for the asymptomatic and patient volunteers, respectively. At the other TEs, the median relative residuals range from 2.4% to 8.1% for the asymptomatic volunteer, and from 2.5% to 7.7% for the patient volunteer. Visually, the residual maps at TE_2-TE_6 exhibit some outlines of mostly bone, tendon or muscle. Yet, the signal content in these residuals remains markedly small compared to the to the signal contained in the NMF components

(except for component 1 at TE_3-TE_6 due to the rapidly decaying signal characteristic, and component 3 at out-of-phase echoes TE_3 and TE_6).

To further analyze the NMF residuals, a comparison to the residuals from the bi-exponential T_2* mapping for the same asymptomatic and patient volunteers as in **Figure S*8*** and **Figure S*9*** is included in **Figure S10**.

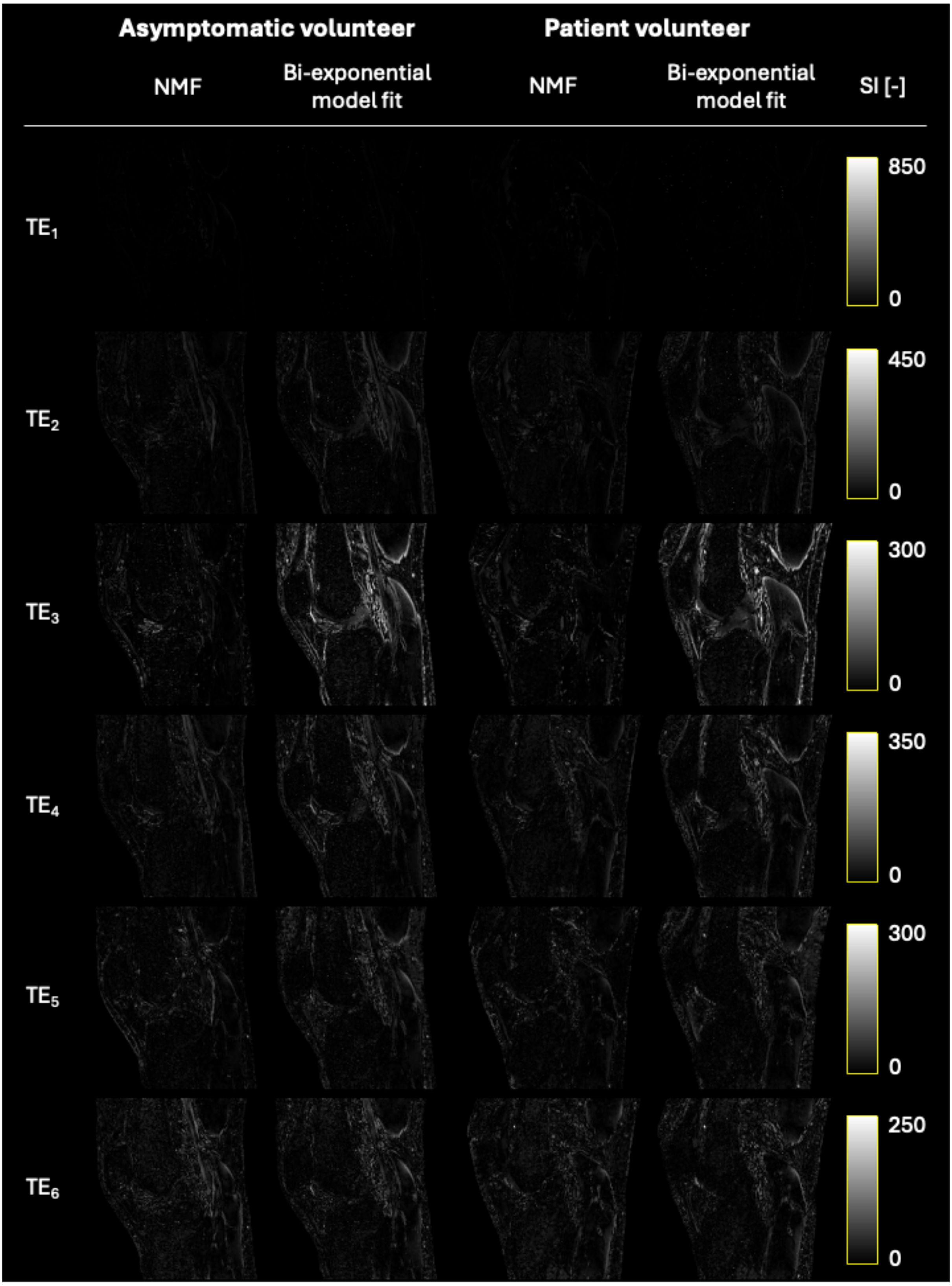

Figure S10. *Residual analysis of the convexity-constrained NMF and bi-exponential T_2* mapping applied to the UTE dataset of a 23-year-old female asymptomatic volunteer (left side) and a 46-year-old female patient volunteer with pivot-shift injury (right side). Residual maps of the NMF are shown next to the corresponding residual maps of the bi-exponential model fit (columns) for each echo time (TE) (rows). The upper limit of the corresponding unitless signal intensity (SI) scale was set to the rounded median of the maximum signal intensity values of the component intensities at the given TE.*

For both subjects, the residuals of the convexity-constrained NMF and bi-exponential T_2* mapping are overall very similar, except at TE_3 (out-of-phase) which shows an increased residual signal for the bi-exponential mapping in both subjects. The residual maps of both methods also display some remaining anatomical structure, particularly at tissue boundaries. At TE_1, the median relative residuals (with respect to the total acquired signal) are 0.2% for both volunteers for the bi-exponential model fit. At the other TEs, the median relative residuals for the bi-exponential model fit range from 4.9% to 9.9% (asymptomatic) and from 5.8% to 9.6% (patient). These values are relatively higher than for the convexity-constrained NMF. However, as previously mentioned, the mix of in-phase, out-of-phase and intermediate TEs may have affected the bi-exponential T_2* mapping as no off-resonance components were considered in the model-based analysis.

S4. Rank analysis

Figure S11 and **Figure S12** show the results of the convexity-constrained NMF applied to the large dataset from the pilot study for rank k equal to 3, 4 and 5. **Figure S11** shows the basis functions of the three considered decompositions, while **Figure S12** displays the corresponding weight maps.

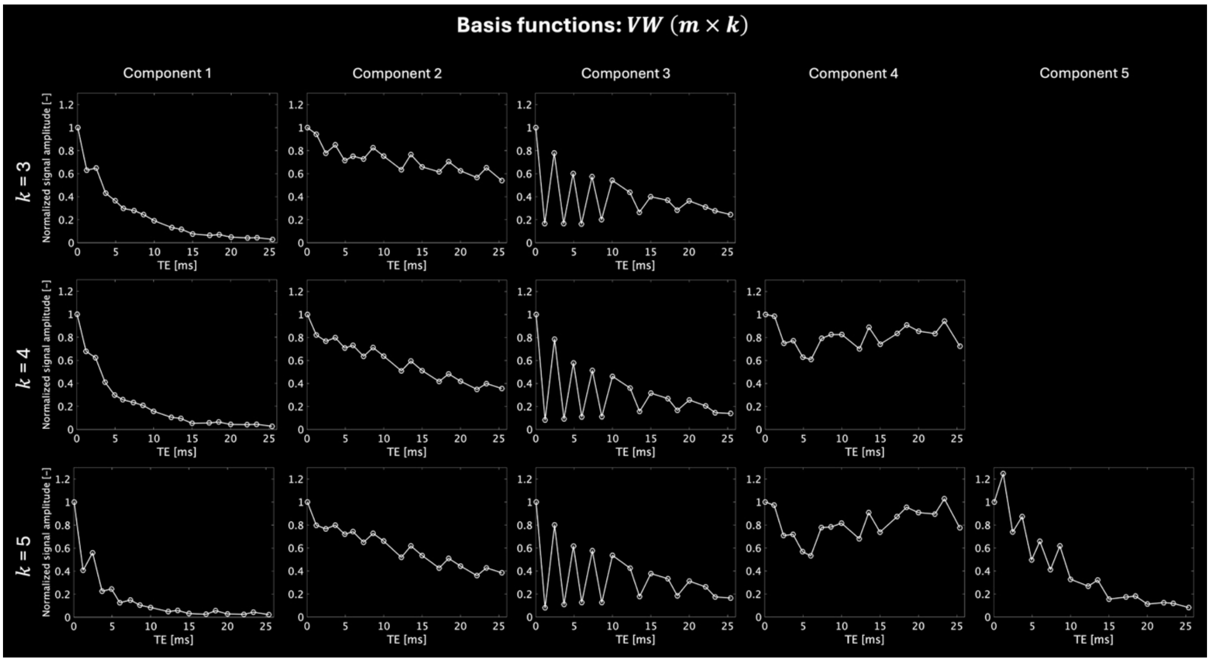

Figure S11. *Basis functions resulting from the convexity-constrained NMF applied to the large dataset from the pilot study for a rank k of 3, 4 and 5. The shown basis functions are normalized to the maximal signal intensity (at TE_1).*

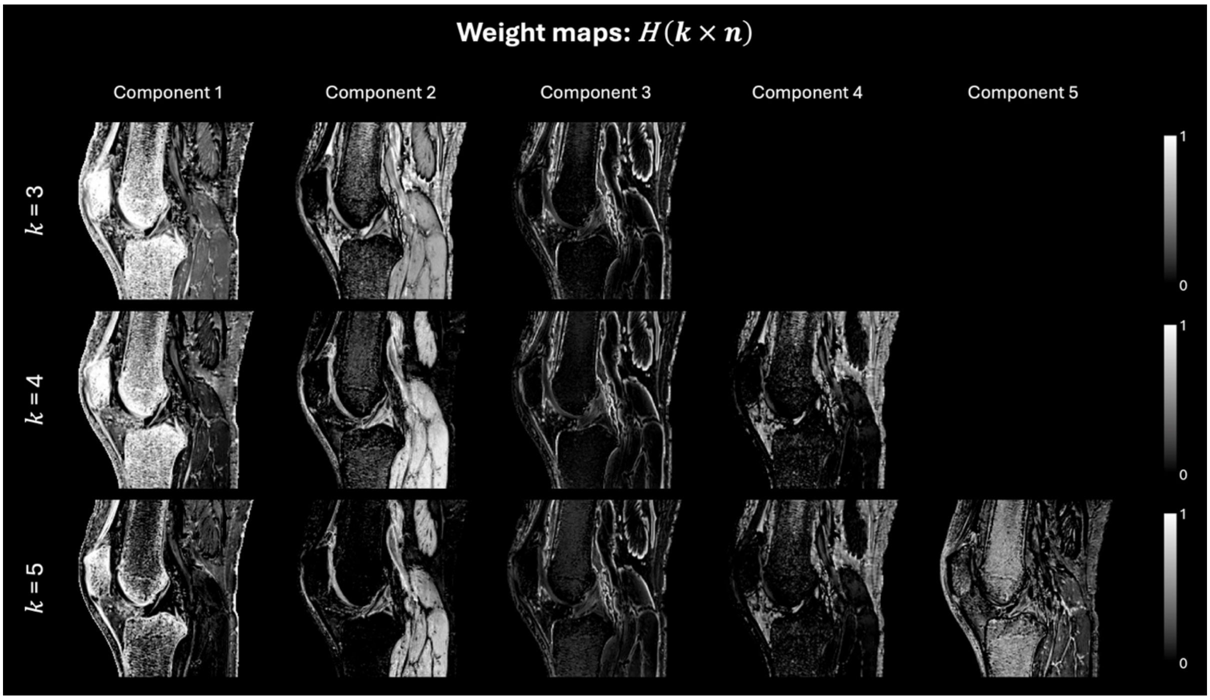

Figure S12. *Weight maps resulting from the convexity-constrained NMF applied to the large dataset from the pilot study for a rank k of 3, 4 and 5. The weight maps are normalized to sum up to 1.*

Across all three decompositions, component 3 remains fairly constant, as observed for both the basis functions and weights maps. For $k = 3$, component 2 highlights both fatty tissues and tissues previously characterized with long T_2* relaxation behavior (e.g., muscle). Moreover, component 1 also exhibits some signal in muscle tissue. For $k = 5$, components 3 and 4 have very similar basis functions and weight maps as in the decomposition for $k = 4$. The weight map of the additional component (component 5) mostly highlights bone and subcutaneous tissue, which were previously largely depicted in the weight maps of components 1 and 2 obtained for $k = 4$.

S5. References

1. Ruschke S, Zoellner C, Boehm C, Diefenbach MN, Karampinos DC. Chemical Shift Encoding-Based Water-Fat Separation. In: 2022:391-418. doi:10.1016/B978-0-12-822726-8.00025-7
2. Pasanta D, Htun KT, Pan J, et al. Magnetic Resonance Spectroscopy of Hepatic Fat from Fundamental to Clinical Applications. *Diagnostics*. 2021;11(5):842. doi:10.3390/diagnostics11050842
3. Rothe M, Riedel S, Slawig A, et al. Fast 3D UTE in vivo T1 and T∗2 mapping of fast relaxing knee tissues at 3T. *Magn Reson Med*. 2026;95(2):693-705. doi:10.1002/mrm.70099
4. Chan KS, Lee H, Ma Y, et al. GACELLE: GPU-accelerated tools for model parameter estimation and image reconstruction. *ArXiv*. Published online November 27, 2025. http://www.ncbi.nlm.nih.gov/pubmed/41356069
